# Non-Malleable Code in the Split-State Model

**DOI:** 10.3390/e24081038

**Published:** 2022-07-28

**Authors:** Divesh Aggarwal, Marshall Ball, Maciej Obremski

**Affiliations:** 1 Department of Computer Science and Center for Quantum Technologies, National University of Singapore, Singapore 119077, Singapore; 2 Courant Institute of Mathematics, New York University, New York, NY 10012, USA; marshall@cs.nyu.edu; 3 Center for Quantum Technologies, National University of Singapore, Singapore 119077, Singapore; obremski.math@gmail.com

**Keywords:** non-malleable codes, split-state NMC, strong NMC, super NMC, NMC compilers

## Abstract

Non-malleable codes are a natural relaxation of error correction and error detection codes applicable in scenarios where error-correction or error-detection is impossible. Over the last decade, non-malleable codes have been studied for a wide variety of tampering families. Among the most well studied of these is the split-state family of tampering channels, where the codeword is split into two or more parts and each part is tampered with independently. We survey various constructions and applications of non-malleable codes in the split-state model.

## 1. Introduction

Motivated by applications in tamper-resilient hardware, Dziembowski, Pietrzak, and Wichs [1] introduced non-malleable codes as a natural generalization of error correction and error detection codes.

The error correction and the error detection codes are the most basic objects in the codes theory. They do, however, have significant drawbacks, which makes them unsuitable for the applications to tamper-resilient cryptography. In the case of error correction codes, the message can be retrieved as long as only a limited number of positions of the codeword have been flipped; however, it is hard to find a scenario where an adversary would limit himself to flipping only a few positions when given access to the whole codeword. The error detection codes face a different interesting challenge, namely whatever tampering limitations we impose on the adversary (be it polynomial time, bounded memory or some structure limitations like split-state), the adversary can not be allowed to overwrite the codeword. Overwriting a codeword with another valid pre-computed codeword makes the detection of tampering clearly impossible. However, overwrites are quite simple attacks, and the adversary wipes the memory of the device, and uploads some new data. While this attack seems irrational, there are scenarios when partial overwrites are realistic attacks on the scheme (Those attacks often allow the adversary to gradually learn the underlying secret key. They are especially prevalent in the natural scenarios where the adversary gets to tamper with the device multiple times.). Naturally, we would like to allow for a wide spectrum of attacks including overwrite attacks.

Motivated by this, Dziembowski, Pietrzak, and Wichs [1] considered a notion of non-malleable codes (NMC). It was a weakening of detection/correction codes based on the concept of *non-malleability* introduced by [2].

The model is very natural and clean. We start with the message *m*, we encode it Enc(m)=c, and then we store the encoding on the device; the adversary picks any adversarial function t∈T (where T is a class of tampering channels), a codeword is tampered to $c^{\prime }=t\left(c\right)$, and, after decoding, we obtain $Dec\left(t\left(c\right)\right)=m^{\prime }$. In the *error-correction* codes, we would like $m^{\prime }=m$; in the *error-detection* codes, we would like $m^{\prime }=m$ or $m^{\prime }=⊥$ (where ⊥ is a special symbol denoting detection of tampering). As we already discussed, if the family of channels T contains constant functions, then neither correction nor detection is possible. There is, however, a meaningful definition that can be considered here. Non-malleable code against the family of channels T guarantees that, after the tampering above, $m^{\prime }=m$ or $m^{\prime }$ is completely independent of *m*; for instance, $m^{\prime }=m+1$ is not possible.

Dziembowski, Pietrzak, and Wichs formalized this notion using the simulation paradigm: the output of the experiment can be simulated by a simulator that depends only on the adversarial channel *t* (and not the message *m*), and is allowed to output a special symbol same which is replaced by the encoded message *m*.

**Definition** **1** (Non-malleable codes [1]). *A pair of randomized (Ref. [1] defined Dec to be deterministic; however, here we allow decoding to be randomized; it is not clear if randomized and deterministic decoding are equivalent; in particular, no strong separations are known.) algorithms, (Enc $:{\left\{0,1\right\}}^{k}\to {\left\{0,1\right\}}^{n},\mathrm{Dec}:{\left\{0,1\right\}}^{n}\to {\left\{0,1\right\}}^{k})$, is an ε-non-malleable code with respect to a family of channels $T\subseteq \{f:{\left\{0,1\right\}}^{n}\to {\left\{0,1\right\}}^{n}\}$, if the following properties hold:*

*1.*  *(Correctness)*
  *For every message $m\in {\left\{0,1\right\}}^{k}$,*
  Pr[Dec(Enc(m)=m]=1
  *, where the probability is over the randomness of the encoding and decoding procedures.*
*2.*  *(Security)*
  *For every t∈T, there is a random variable $D_{t}$ supported on ${\left\{0,1\right\}}^{k}\cup \left\{same\right\}$ that is independent of the randomness in Enc,Dec, such that, for every message $m\in {\left\{0,1\right\}}^{k}$*
  $$(\mathrm{Dec}\left(t\left(\mathrm{Enc}\left(m\right)\right)\right)\approx_{\varepsilon }\mathrm{Copy}\left(D_{t},m\right)),$$
  *where $\approx_{\varepsilon }$ denotes statistical distance (total variation distance) at most ε, and the function Copy is defined as*
  $$\mathrm{Copy}\left(x,y\right)=\left\{\begin{array}{cc} x & \mathrm{if}\;x\neq same\\ y & \mathrm{if}\;x=same. \end{array}\right.$$

A few years later, Aggarwal, Dodis, Kazana, and Obremski [3] introduced an alternative perspective on non-malleable codes by introducing the notion of non-malleable reductions. To intuitively describe a non-malleable reduction, imagine the scenario discussed earlier, where the message *m* is encoded as a codeword *c*, and *c* is tampered using t∈T into $c^{\prime }=t\left(c\right)$. The tampered codeword $c^{\prime }$ decodes to $m^{\prime }$. A non-malleable reduction from T to G guarantees that $m^{\prime }=g\left(m\right)$, where *g* is a possibly randomized tampering function sampled from G. In particular, if the family of functions G contains only the identity function and all constant functions, then the corresponding non-malleable reduction is a non-malleable code for T.

Motivation: tamper-resilient hardware

The relaxed guarantees of a non-malleable code may seem a bit arbitrary at first glance; however, the object has natural applications in tamper resilient hardware. In the 1990s, high profile side-channel attacks on a number of cryptographic schemes were published that broke security by evaluating the schemes on a sequences of algebraically-related keys [4,5]. A number of ad-hoc solutions for these “related-key attacks” were suggested, and eventually theoretical solutions were proposed by Gennaro, Lysyanskaya, Malkin, Micali, and Rabin [6] as well as Ishai, Prabhakaran, Sahai, and Wagner [7].

In [6], the authors addressed tampering attacks with a solution that assumes a (public) tamper and leakage resilient circuit in conjunction with leakage resilient memory. The justification for using tamper and leakage resilient-hardware was two-fold: (1) leakage-resilience had been addressed far more systematically in the literature and existing approaches could be applied off-the-shelf, (2) because the tamper and leakage resilient circuit was public (in particular, contained no secret keys), it was safer to assume appropriately hardened hardware could be responsibly manufactured; their approach was to sign the contents of memory with a strong signature scheme. Unfortunately, this also makes it infeasible to update the memory without a secret key (which would again need to be protected). In [7], it was shown how to compile a circuit into a tamper-resilient one, building on ideas from secure computation. Unfortunately, the tampering attacks handled by this approach are quite limited, and it has proven difficult to extend their results to more general tampering attacks.

Dziembowski, Pietrzak, and Wichs motivated non-malleable codes as a means of extending the approach of [6]. They considered the same model of a tamper and leakage resilient (public) circuit with leakage-resilient memory, but instead of signing the memory (using a secret key), the memory is encoded with a (public) non-malleable code. This allows the tamper and leakage resilient circuit to update any state in the memory itself by decoding, computing, and re-encoding. In contrast with [6], where a trusted third party holding the secret signing key is needed to sign new memory contents, this achieves non-malleability for stateful functionalities. The downside is that, while a strong signature scheme is resilient to arbitrary polynomial time tampering attacks, efficient non-malleable codes (because they are public) have no hope of handling such attacks (See Feasibility below, as well as Section 7 for more details).

Feasibility

It is not difficult to see that non-malleable codes only exist for restricted classes of channels T: otherwise, one can always consider the channel that decodes the message, flips a bit, and re-encodes the resulting message. Thus, the natural question to ask is against which classes of tampering channels is it possible to build non-malleable codes. As a first result, Dziembowski, Pietrzak, and Wichs gave an efficient, explicit construction of a non-malleable code against channels that can tamper each codeword bit independently (so-called “bitwise tampering”). They additionally provided a non-constructive argument that ε-non-malleable codes exist for any class of channels that is not too big, i.e., loglog|T|<n−2log1/ε. They left constructing explicit codes for larger, richer classes of channels as an open problem.

Split-state non-malleable codes

A well-studied class of tampering functions is the 2-split-state model where the codeword consists of two states *L* and *R*, and the adversary tampers with each of these states independently. This is a very large class of tampering channels that, in particular, includes the bitwise tampering family of channels mentioned above. We now sketch the landscape of this area and particularly summarize the results on 2-split-state NMCs in Table 1. In [1], in addition to introducing non-malleable codes, the authors also introduced a model of tampering called the *t*-split-state model, where the codeword consists of *t* independently tamperable states. They give the first NMC constructions in the *n*-split-state model (We already mentioned this result above as a non-malleable code against bitwise tampering. We mention it again just to emphasize that bitwise tampering is a special case of split-state tampering) (where *n* is the codeword length) and the 2-split-state model (using random oracles). Dziembowski, Kazana, and Obremski [8] provided the first construction of 2-split-state NMCs without any assumptions. Their construction enabled encoding of 1-bit messages and used two-source extractors. The first NMC in the 2-split-state model for *k*-bit messages was given by Aggarwal, Dodis, and Lovett [9], which used inner product extractors with tools from additive combinatorics. In [10], Cheraghchi and Guruswami studied the optimal rate of the non-malleable codes for various tampering families, where the rate of a code is defined as $\frac{\mathrm{message}\;\mathrm{length}}{\mathrm{codeword}\;\mathrm{length}}$. In particular, they showed that the optimal achievable rate for the *t*-split-state family is 1−1/t. Note that, in the split-state tampering model, having as few states as possible is most desirable, with two states being the best achievable. By the above result, the best possible rate for the 2-split-state model is therefore $\frac{1}{2}$. A long series of works (Other works have considered non-malleable codes in models other than the 2-split-state model or under computational assumptions [11,12,13,14,15,16,17,18,19,20,21,22,23,24,25,26]). Refs. [3,24,27,28,29,30,31,32,33,34,35] have made significant progress towards achieving this rate. We now discuss some of these results. The work of Cheraghchi and Guruswami [27] gave the first optimal rate non-malleable code in the *n*-split-state model (where *n* is the codeword length). More importantly, this work introduced non-malleable two-source extractors and demonstrated that these special extractors can be used to generically build 2-split-state NMCs. This connection has led to several fascinating works [28,29,30,31] striving to improve the rate and number of states of non-malleable codes. Most notably, Chattopadhyay and Zuckerman [28] built a 10-state NMC with a constant rate, making this the first constant rate construction with a constant number of states. They achieve their result by first building a non-malleable extractor with 10 sources and then using the connection due to [10] to obtain the corresponding non-malleable code. The work of Kanukurthi, Obbattu, and Sekar [32] used seeded extractors to build a compiler that transforms a low rate non-malleable code into one with high rate and, in particular, obtained a rate 1/3, 4—state non-malleable code. This was subsequently improved to three states in the works of Kanukurthi, Obbattu, and Sekar [33] as well as Gupta, Maji, and Wang [24]. Li [31] obtained 2-split-state NMC with rate $O(\frac{\log\log\log(1/\varepsilon )}{\log\log(1/\varepsilon )})$ (where ε is the error). Particularly, this gave a rate of O(loglog(n)/log(n)), for negligible error $\varepsilon =2^{-\Omega (n)}$, and a constant rate for constant error, making this the first constant rate scheme in the 2—split-state model. The concept of non-malleable reductions due to [3] was used to build the first constant rate NMC with negligible error in the 2-split-state model [34]. In a recent work, Ref. [35], it was shown that the construction from [32] (with rate 1/3) is actually non-malleable even against two split-state tampering (and hence is nearly an optimal rate construction for two split-state tampering).

Applications of split-state non-malleable codes

The split-state tampering model is a very natural model. In particular, there are cryptographic settings where the separation of states is natural, like in secret sharing or in multiparty computation (MPC) scenarios. Non-malleable codes in the split-state model have found many applications in achieving security against physical (leakage and tampering) attacks [1,36], domain extension of encryption schemes [37,38], non-malleable commitments [39], non-malleable secret sharing [40,41,42,43], non-malleable oblivious transfer [44], and privacy amplification [45]. We discuss the application to non-malleable commitments in more detail in Section 8.

Additionally, non-malleable codes in the split-state model have found many applications in the construction of non-malleable codes against other important and natural tampering families, as mentioned below:

- Decision tree tampering ([46]): each tampered output symbol is a function of a small polynomial number of (adaptively chosen) queries to codeword symbols.
- Small-depth circuit tampering ([46,47]): the tampered codeword is produced by a boolean circuit of polynomial size and nearly logarithmic depth.
- (Bounded) Polynomial-size circuit tampering ([48]): the tampered codeword is produced by circuit of bounded polynomial size, $n^{d}$ for some constant *d*, where *n* is the codeword length.
- Continuous NMCs ( [16]): the tampering is still split-state, but the adversary is allowed to tamper repeatedly until the tampering is detected.

The applications to decision tree tampering, small-depth circuits, and polynomial size circuit tampering are discussed in Section 7.

### Organization of the Paper

- Section 2 contains preliminaries and definition of non-malleable reductions, and the reader may refer to it when required;
- Section 3 contains a gentle introduction to different variants of non-malleable codes and their properties such as secret sharing and leakage-resilience.
- In Section 4, we give the first, and arguably the simplest, construction of non-malleable codes in the split-state model [9]. The simplicity made it a particularly useful tool for several follow-up works that required non-standard properties from the underlying non-malleable code.
- In Section 5, we briefly mention two-source non-malleable extractors, and their connection to non-malleable codes in the split-state model, as well as to other cryptographic primitives.
- In Section 6, we give an overview of the rate amplification technique from [32,33,34,35] that gives an almost optimal rate non-malleable code in the split-state model.
- In Section 7, we survey some of the applications of split-state non-malleable codes to constructing non-malleable codes against computationally bounded tampering classes. In particular, we give an overview of the techniques in the following works: Ref. [46] for small-depth decision trees, Ref. [47] for small-depth circuit tampering, and Ref. [48] for polynomial size circuit tampering.
- In Section 8, we give a construction of a non-malleable commitment scheme due to [39] that is one of the most important applications of non-malleable codes in the split-state model.

## 2. Preliminaries

### 2.1. Notation and Mathematical Preliminaries

For a set *T*, let $U_{T}$ denote a uniform distribution over *T*, and, for an integer *ℓ*, let $U_{\ell }$ denote uniform distribution over *ℓ* bit strings. We say that b=a±δ if a−δ≤b≤a+δ. For any random variable *A* and any set A, we denote ${A|}_{A\in A}$ to be the random variable $A^{\prime }$ such that

$$\forall a,\;\mathrm{Pr}\left[A^{\prime }=a\right]=\mathrm{Pr}\left[A=a\;|\;A\in A\right]\;.$$

The *statistical distance* between two random variables A,B is defined by

$$\Delta \left(A\;;\;B\right)=\frac{1}{2}\sum_{v}\left|\mathrm{Pr}[A=v]---\mathrm{Pr}[B=v]\right|\;.$$

We use $A\approx_{\varepsilon }B$ as shorthand for Δ(A,B)≤ε.

**Lemma** **1.** *For any function α, if Δ(A;B)≤ε, then Δ(α(A);α(B))≤ε.*

The *min-entropy* of a random variable *W* is $H_{\infty }$$\left(W\right)\overset{\mathrm{def}}{=}-\log\left(\max_{w}\mathrm{Pr}\left[W=w\right]\right)$, and the *conditional* min-entropy of *W* given *Z* is $H_{\infty }\left(W|Z\right)\overset{\mathrm{def}}{=}-\log\left({\mathrm{error}}_{z\leftarrow Z}\max_{w}\mathrm{Pr}\left[W=w|Z=z\right]\right)$.

**Definition** **2.** *We say that a function $\mathrm{Ext}\;:F^{n}\times F^{n}\to F$ is a (k,ε)-*2-source extractor*if, for all independent sources $X,Y\in F^{n}$ such that min-entropy $H_{\infty }$$\left(X\right)+H_{\infty }\left(Y\right)\geq k$, we have $\left(Y,\mathrm{Ext}\left(X,Y\right)\right)\approx_{\varepsilon }\left(Y,U_{m}\right)$, and $\left(X,\mathrm{Ext}\left(X,Y\right)\right)\approx_{\varepsilon }\left(X,U_{m}\right)$.*

**Lemma** **2.** *For all positive integers n and any finite field F, and for all ε>0, the inner product function $\langle \cdot ,\cdot \rangle :F^{n}\times F^{n}\to F$ is an efficient $\left(\left(n+1\right)\log|F|+2\log\left(\frac{1}{\varepsilon }\right),\varepsilon \right)$ 2-source extractor.*

In particular, for *n* being an integer multiple of *m*, and interpreting elements of ${\left\{0,1\right\}}^{m}$ as elements from $F_{2^{m}}$ and those in ${\left\{0,1\right\}}^{n}$ to be from ${\left(F_{2^{m}}\right)}^{n/m}$, we have that, for any ε>0, there exists an efficient $\left(n+m+2\log\left(\frac{1}{\varepsilon }\right),\varepsilon \right)$ 2-source extractor $\mathrm{Ext}:{\left\{0,1\right\}}^{n}\times {\left\{0,1\right\}}^{n}\to {\left\{0,1\right\}}^{m}$.

The following is a definition of an ε-almost universal hash function.

**Definition** **3.** *A function $C:{\left\{0,1\right\}}^{s}\times {\left\{0,1\right\}}^{n}\to {\left\{0,1\right\}}^{t}$ is called an*ε-almost universal hash function *if, for any $x,y\in {\left\{0,1\right\}}^{n}$ such that x≠y,*

$$\underset{R\leftarrow {\left\{0,1\right\}}^{s}}{\mathrm{Pr}}\left(C\left(R,x\right)=C\left(R,y\right)\right)\leq \varepsilon$$

The following is a standard construction of a polynomial evaluation ε-universal hash function. The parameters are from [51].

**Lemma** **3.** *For any n,t>2logn, there exists an efficiently computable $2^{-t/2}$-almost universal hash function $C:{\left\{0,1\right\}}^{s}\times {\left\{0,1\right\}}^{n}\to {\left\{0,1\right\}}^{t}$ with s=2t.*

**Lemma** **4** (Lemma 4 of [52], Lemma 9 of [3]). *Let A,B be independent random variables and consider a sequence $V_{1},\ldots ,V_{i}$ of random variables, where for some function ϕ, $V_{i}=\phi_{i}\left(C_{i}\right)=\phi \left(V_{1},\ldots ,V_{i-1},C_{i}\right)$ with each $C_{i}\in \left\{A,B\right\}$. Then, A and B are independent conditioned on $V_{1},\ldots ,V_{i}$. That is, $I(A;B|V_{1},\ldots ,V_{i})=0$.*

### 2.2. Non-Malleable Codes and Reductions

In [3], the notion of non-malleable codes w.r.t. to a tampering family F (see [1]) was generalized to a more versatile notion of *non-malleable reductions* from F to G. The following definitions are taken from [3].

**Definition** **4**(non-malleable reduction). *Let $F\subset A^{A}$ and $G\subset B^{B}$ be some classes of functions (which we call* manipulation *functions). We will write:*

(F⇒G,ε)

*and say* F reduces to G*, if there exist an efficient randomized* encoding *function E:B→A, and an efficient deterministic *decoding* function D:A→B, such that, (a) for all x∈B, we have D(E(x))=x, and (b) for all f∈F, there exists G such that, for all x∈B,*

(1) $$\Delta \left(D(f(E(x)))\;;\;G(x)\right)\leq \varepsilon ,$$

*where G is a distribution over G, and G(x) denotes the distribution g(x), where g←G.*

*The pair (E,D) is called (F,G,ε)-non-malleable reduction.*

Intuitively, (F,G,ε)-non-malleable reduction allows one to encode a value *x* by y←E(x), so that tampering with *y* by $\underline{y}=f\left(y\right)$ for f∈F gets “reduced” (by the decoding function $D\left(\underline{y}\right)=\underline{x}$) to tampering *with x itself* via some (distribution over) g∈G.

In particular, the notion of *non-malleable code* w.r.t. F, is simply a reduction from F to the family of “trivial manipulation functions” ${\mathrm{NM}}_{k}$ defined below.

**Definition** **5.** Let ${\mathrm{NM}}_{k}$ denote the set of *trivial manipulation functions* on *k*-bit strings, which consists of the identity function I(x)=x and all constant functions $f_{c}\left(x\right)=c$, where $c\in {\left\{0,1\right\}}^{k}$.

We say that a pair (E,D) defines an *(F,k,ε)-non-malleable code*, if it defines a $\left(F,{\mathrm{NM}}_{k},\varepsilon \right)$-non-malleable reduction.

The utility of non-malleable reductions comes from the following natural composition theorem that was shown in [3], which allows for gradually making our tampering families simpler and simpler, until we eventually end up with a non-malleable code (corresponding to the trivial family ${\mathrm{NM}}_{k}$).

**Theorem** **1** (Composition)**.**
*If $\left(F\Rightarrow G,\varepsilon_{1}\right)$ and $\left(G\Rightarrow H,\varepsilon_{2}\right)$, then $\left(F\Rightarrow H,\varepsilon_{1}+\varepsilon_{2}\right)$.*

We will also need the following trivial observation.

**Observation** **1** (Union)**.**
Let (E,D) be an (F,H,ε) and a $\left(G,H,\varepsilon^{\prime }\right)$ non-malleable reduction. Then, (E,D) is an $\left(F\cup G,H,\max\left(\varepsilon ,\varepsilon^{\prime }\right)\right)$ non-malleable reduction.

Useful Tampering Families. We define several natural tampering families we will use in this work. For this, we first introduce the following “direct product” operator on tampering families:

**Definition** **6.** *Given tampering families $F\subset A^{A}$ and $G\subset B^{B}$, let F×G denote the class of functions h from ${\left(A\times B\right)}^{A\times B}$ such that*

$$h\left(x\right)=h_{1}\left(x_{1}\right)∥h_{2}\left(x_{2}\right)$$

*for some $h_{1}\in F$ and $h_{2}\in G$ and $x=x_{1}∥x_{2}$, where $x_{1}\in A,x_{2}\in B$.*

*We also let $F^{1}:=F$, and, for t≥1, $F^{t+1}:=F^{t}\times F$.*

We can now define the following tampering families:

- $S_{n}={\left({\left\{0,1\right\}}^{n}\right)}^{{\left\{0,1\right\}}^{n}}$ denotes the class of *all* manipulation functions on *n*-bit strings.
- $S_{n,p}={\left(F_{p}^{n}\right)}^{F_{p}^{n}}$ denotes the class of *all* manipulation functions on $F_{p}^{n}$.
- Given t>1, $S_{n,p}^{t}$ denotes the tampering family in the *t-split-state model*, where the attacker can apply *t* arbitrarily correlated functions $h_{1},\ldots ,h_{t}$ to *t* separate, parts of memory each in $F_{p}^{n}$ (but, of course, each $h_{i}$ can only be applied to the *i*-th part individually).
- Given a prime *p*, ${\mathrm{AFF}}_{p}$ denotes the class of all affine functions parametrized by $a,b\in F_{p}$ such that $f_{a,b}\left(x\right):=ax+b$ for all $x\in F_{p}$.

### 2.3. Basic Techniques

The following is a simple result from [9] that says that, if two pairs of random variables $\left(X_{1},X_{2}\right),\left(Y_{1},Y_{2}\right)$ are statistically close to each other, then $X_{1}$ conditioned on $X_{2}$ is statistically close to $Y_{1}$ conditioned on $Y_{2}$.

**Lemma** **5.** *Let $X_{1},Y_{1}\in A_{1}$, and $Y_{1},Y_{2}\in A_{2}$ be random variables such that $\Delta (\left(X_{1},X_{2}\right);\left(Y_{1},Y_{2}\right))\leq \varepsilon$. Then, for any non-empty set $A^{\prime }\subseteq A_{1}$, we have*

$$\Delta (X_{2}\;|\;X_{1}\in A^{\prime }\;;\;Y_{2}\;\left|\;Y_{1}\in A^{\prime }\right)\leq \frac{2\varepsilon }{\mathrm{Pr}(X_{1}\in A^{\prime })}\;.$$

The following is a variant of a similar simple lemma from [8,9]. The proof is just a simple application of triangle inequality.

**Lemma** **6.** *Let S be some random variable distributed over a set S, and let $S_{1},\ldots ,S_{j}$ be a partition of S. Let ϕ:S→T be some function, and let $D_{1},\ldots ,D_{j}$ be some random variables over the set T. Assume that, for all 1≤i≤j,*

$$\Delta \left({\phi \left(S\right)|}_{S\in S_{i}}\;;\;D_{i}\right)\leq \varepsilon_{i}.$$

*Then,*

$$\Delta \left(\phi (S)\;;\;D\right)\leq \sum \varepsilon_{i}\mathrm{Pr}\left[S\in S_{i}\right]\;,$$

*for some random variable D∈T such that for all d $\mathrm{Pr}\left[D=d\right]=\sum_{i}\mathrm{Pr}\left[S\in S_{i}\right]\cdot \mathrm{Pr}\left[D_{i}=d\right]$.*

**Lemma** **7.** *Let F be a finite field. Let $X=(X_{1},X_{2})\in F\times F$ be a random variable. Assume that, for all a∈F not both zero, $\Delta (X_{1}+aX_{2}\;;\;U_{F})\leq \varepsilon$. Then, $\Delta \left(\left(X_{1},X_{2}\right)\;;\;U_{F_{p}},X_{2}\right)\leq \varepsilon {\left|F\right|}^{2}$.*

**Lemma** **8.** *Let X∈F be a random variable. Assume that $\Delta (X\;;\;U_{F})\geq \varepsilon$. Then, if $X^{\prime }$ is an i.i.d copy of X, then*

$$\mathrm{Pr}\left[X=X^{\prime }\right]\geq \frac{1+\varepsilon^{2}}{\left|F\right|}\;.$$

**Lemma** **9.** *Let $Z=\left(X,Y\right)\in F^{n}\times F^{n}$ be a random variable, and let $Z^{\prime }=\left(X^{\prime },Y^{\prime }\right)$ be an i.i.d copy of Z. Then,*

$$\mathrm{Pr}\left[\langle X,Y\rangle =\langle X^{\prime },Y^{\prime }\rangle \right]\leq \mathrm{Pr}\left[\langle X,Y\rangle =\langle X^{\prime },Y\rangle \right].$$

## 3. Basic Properties and Variants of Non-Malleable Codes and Continuous Non-Malleable Codes

In this section, we will discuss various basic properties of the 2−split state non-malleable codes, as well as numerous variants of their definitions.

### 3.1. A Few Examples

As a warm up, we will go through few basic examples of codes that are not non-malleable.

**Example** **1.** *To encode m∈F, we pick L∈F and R∈F uniformly random such that L+R=m.*

Above clearly is not a non-malleable code: pick $\underline{L}=L+1$ and $\underline{R}=R$; then, $\mathrm{Dec}\left(\underline{L},\underline{R}\right)=\mathrm{Dec}\left(L,R\right)+1$; we have changed the message, but the message is not independent of the original message.

**Example** **2.** *To encode $m\in F_{p}$, pick $L,R\in F_{p}^{n}$ uniformly random such that 〈L,R〉=m, where 〈.,.〉 stands for the inner product over $F_{p}$.*

Again, the attack is quite simple: pick a∉{0,1} and let $\underline{L}=a\cdot L$, $\underline{R}=R$, then $\mathrm{Dec}\left(\underline{L},\underline{R}\right)=a\cdot \mathrm{Dec}\left(L,R\right)$. Again, the message has changed but remained strongly correlated with the original message. The above attack depends on a∉{0,1}; however, over $F_{2}$, we will not have any other option. Thus, maybe let us consider the following code:

**Example** **3.** *To encode $m\in F_{2}$, pick $L,R\in F_{2}^{n}$ uniformly at random such that 〈L,R〉=m, where 〈.,.〉 stands for the inner product over $F_{2}$.*

Sadly again, there is a simple attack; let $\underline{L}$ (and $\underline{R}$ respectively) be equal to *L* (and *R* respectively) on all positions except the last, we will set the last position to ${\left(\underline{L}\right)}_{n}=1$ (and ${\left(\underline{R}\right)}_{n}=1$). Now, with probability $\frac{3}{4}$, we have $\mathrm{Dec}\left(\underline{L},\underline{R}\right)=\mathrm{Dec}\left(L,R\right)⊕1$, and with probability $\frac{1}{4}$ we have $\mathrm{Dec}\left(\underline{L},\underline{R}\right)=\mathrm{Dec}\left(L,R\right)$. This can not be a non-malleable code, as [8] showed for single bit message (We also assume that Dec≠⊥, i.e., there are no invalid codewords): if we can flip the output of the Decoder with probability greater then $\frac{1}{2}$ (plus some non-negligible factor), then the code can not be non-malleable.

### 3.2. Secret Sharing

We will show that the 2—split state non-malleable code has to be a 2 out of 2 secret sharing. Let $m_{0},m_{1}$ be two messages and let $\mathrm{Enc}\left(m_{0}\right)=X_{0},Y_{0}$ and $\mathrm{Enc}\left(m_{1}\right)=X_{1},Y_{1}$. If given $X_{i}$, we could guess *i*, and we would be able to tamper the codewords in a way that $\mathrm{Dec}\left({\underline{X}}_{0},{\underline{Y}}_{0}\right)=a_{0}$ and $\mathrm{Dec}\left({\underline{X}}_{1},{\underline{Y}}_{1}\right)=a_{1}$, where $a_{0},a_{1}$ are two fixed distinct messages (different than $m_{0},m_{1}$). This clearly breaks non-malleability since the original messages $m_{0},m_{1}$ are not preserved, but the messages after tampering are correlated with the original messages: tampered message is $a_{i}$ if and only if the original message was $m_{i}$. This conveys the main intuition: if the message is not preserved, then tampered message should not reveal the original message.

Let us construct the above-mentioned attack: find $\ell_{0},\ell_{1},r,a_{0}\neq a_{1}$ such that $\mathrm{Dec}\left(\ell_{0},r\right)=a_{0}$ and $\mathrm{Dec}\left(\ell_{1},r\right)=a_{1}$ (we know they must exist else *r* alone would determine the output of the decoder, and we could carry on the same attack on the right state). Now for the tampering: we will completely overwrite the right state $Y_{i}\to r$, and, given $X_{i}$, if we think i=0, we will tamper with $X_{i}\to \ell_{0}$; if we think that i=1, then we tamper with $X_{i}\to \ell_{1}$; this gives the desired result.

To be more precise, we recall the theorem from [20].

**Lemma** **10** (from [20])**.** *If (Enc,Dec) is an ε— non-malleable code, then for any two messages $m_{0},m_{1}$, and for $\mathrm{Enc}\left(m_{0}\right)=X_{0},Y_{0}$ and $\mathrm{Enc}\left(m_{1}\right)=X_{1},Y_{1}$, we obtain:*

$$\begin{array}{c} X_{0}\approx_{2\varepsilon }X_{1}\;\;and\;\;Y_{0}\approx_{2\varepsilon }Y_{1} \end{array}$$

### 3.3. Leakage-Resilience

Thus far, we only discussed active adversary that tampers the states. It is natural to consider its weaker version: a passive adversary.

A long time ago, we thought of a cryptographic device as a box that holds a secret key and has a strictly defined interface, and the attacker is only allowed to use that well-defined input/output interface. However, no device is a true blackbox; it consumes electricity, emits electromagnetic radiation, and has a heat signature and a running time; those values were not predicted in the clean blackbox-security model, and thus the first wave of *passive attacks* was born. Now the adversary could exploit side-channel information like the one mentioned above and with its help break the security of the device. We often refer to such side information as *leakage*, and the adversary that exploits it as a *passive adversary*.

We can start with the following theorem:

**Theorem** **2** (from [15])**.** *Let k≥3, and let ε<1/20. Let $\mathrm{Enc}:{\left\{0,1\right\}}^{k}\to {\left\{0,1\right\}}^{n}\times {\left\{0,1\right\}}^{n}$, $\mathrm{Dec}:{\left\{0,1\right\}}^{n}\times {\left\{0,1\right\}}^{n}\to {\left\{0,1\right\}}^{k}$ be an ε—non-malleable code in the split state model. For every set $A,B\subset {\left\{0,1\right\}}^{n}$ and every message $m_{0},m_{1}\in {\left\{0,1\right\}}^{k}$,*

$$|\mathrm{Pr}\left(\mathrm{Enc}\left(m_{0}\right)\in A\times B\right)---\mathrm{Pr}\left(\mathrm{Enc}\left(m_{1}\right)\in A\times B\right)|\leq \varepsilon \;.$$

Before we get to the proof, notice that one can run the above lemma for the following sets: $A\times {\left\{0,1\right\}}^{n}$ and ${\left\{0,1\right\}}^{n}\times B$ and A×B (for the set, the ${\left\{0,1\right\}}^{n}\times {\left\{0,1\right\}}^{n}$ statement is trivial); this means that, given the indicators $1_{A}\left(X_{i}\right)$, $1_{B}\left(Y_{i}\right)$, we can not distinguish between i=0 and i=1 (where $\left(X_{i},Y_{i}\right)$ encode message $m_{i}$). One should remark that, while the above is just a one bit leakage, one can easily leverage it to the arbitrary size *t* leakage at the price of the $2^{t}$ multiplicative penalty in the error; we refer to the similar reasoning below Remark 4.

This is only a mild version of leakage resilience, and we will expand it further in this section.

Below we go over the proof of Theorem 2, and we mention that a similar reasoning forms the core of Remark 4 and Theorem 5.

**Proof.**  We claim that there exist $x,y,z,w\in {\left\{0,1\right\}}^{n}$ such that $m_{0},m_{1},\mathrm{Dec}\left(x,w\right)$, Dec(z,w), and Dec(z,y) are all different from Dec(x,y). Before proving this claim, we show why this implies the given result. Let Enc(m)=X,Y, consider the tampering functions f,g such that f(X)=x if X∈A, and f(X)=z otherwise, and g(Y)=y if Y∈B, and g(Y)=w, otherwise. Thus, for b=0,1, $\mathrm{Dec}\left(\underline{X},\underline{Y}\right)=\mathrm{Dec}\left(x,y\right)$ if and only if $\mathrm{Enc}(m_{b})\in A\times B$. The result then follows from the ε-non-malleability of (Enc,Dec).

Now, to prove the claim, we will use the probabilistic method. Let *U* be uniform in ${\left\{0,1\right\}}^{k}$, and let X,Y←Enc(U). Furthermore, let $W,Z\in {\left\{0,1\right\}}^{n}$ be uniform and independent of X,Y,U. We claim that X,Y,Z,W satisfy the required property with non-zero probability.

It is easy to see that the probability that Dec(X,Y)=U is either of $m_{0}$ or $m_{1}$ is at most $2/2^{k}$. In addition, by Lemma 10, we have that, except with probability 2ε, *X* is independent of *U*. In addition, *W* is independent of *U*. Thus, the probability that Dec(X,W)=U is at most $2\varepsilon +1/2^{k}$. Similarly, the probability that Dec(Z,Y)=U is at most $2\varepsilon +1/2^{k}$. Finally, W,Z are independent of *U*, and so the probability that Dec(Z,W)=U is at most $\frac{1}{2^{k}}$.

Thus, by union bound, the probability that X,Y,Z,W do not satisfy the condition of the claim is at most $\frac{5}{2^{k}}+4\varepsilon \leq \frac{5}{8}+4\varepsilon <1$.

As we already hinted, the above is only a mild version of leakage resilience; for the full version, we would expect, for example, that the decoded message along with the leakage does not reveal anything about the message.

To formalize the above intuitive notion, we first have to recall the original definition from [1]:

**Definition** **7** (Non-Malleable Code from [1])**.** *Let (Enc:M→X×X,Dec:X×X→M∪{⊥}) be an encoding scheme. For f,g:X→X and for any m∈M, define the experiment ${\mathrm{DPWTamper}}_{m}^{f,g}$ as:*

$$\begin{array}{c} {\mathrm{DPWTamper}}_{m}^{f,g}=\left\{\begin{array}{c} (X,Y)\leftarrow \mathrm{Enc}(m),\\ \underline{X}:=f\left(X\right),\underline{Y}:=g\left(Y\right)\\ \underline{m}:=\mathrm{Dec}\left(\underline{X},\underline{Y}\right)\\ output:\underline{m} \end{array}\right\} \end{array}$$

*The above represents the state of the message after tampering. The claim that the message has either not changed or is completely independent of the original message is expressed in a following way: We say that the encoding scheme (Enc,Dec) is ε-DPW-non-malleable in the split-state model if, for every function f,g:X→X, there exists distribution $D^{f,g}$ on M∪{same,⊥} (without the access to the original message) such that, for every m∈M, we have*

$$\begin{array}{c} {\mathrm{DPWTamper}}_{m}^{f,g}\approx_{\varepsilon }\left\{\begin{array}{c} d\leftarrow D^{f,g}\\ ifd=same\;then\;output\;m\\ otherwise\;output\;d. \end{array}\right\} \end{array}$$

In other words, there exists a simulator $D^{f,g}$ that can simulate the tampering experiment; the simulator has no access to the original message: he can only output special symbol same that will be replaced with an original message.

#### Adding Leakage Resilience to Non-Malleable Codes

To add a true leakage resilience, we have few options:

1. Tampering functions might depend on the leakages (e.g., [20,36,46]):
  $$\begin{array}{c} {\mathrm{TamperLeak}}_{m}^{f,g,{\mathrm{Leak}}_{X},{\mathrm{Leak}}_{Y}}=\left\{\begin{array}{c} (X,Y)\leftarrow \mathrm{Enc}(m),\\ \underline{X}:=f\left(X,{\mathrm{Leak}}_{Y}\left(Y\right)\right),\underline{Y}:=g\left(Y,{\mathrm{Leak}}_{X}\left(X\right)\right)\\ \underline{m}:=\mathrm{Dec}\left(\underline{X},\underline{Y}\right)\\ \mathrm{output}:\;\underline{m} \end{array}\right\} \end{array}$$
2. We can also consider outputting the leakage along with the tampered message (e.g., [35,53]):
  $$\begin{array}{c} {\mathrm{TamperLeak}}_{m}^{f,g,{\mathrm{Leak}}_{X},{\mathrm{Leak}}_{Y}}=\left\{\begin{array}{c} (X,Y)\leftarrow \mathrm{Enc}(m),\\ \underline{X}:=f\left(X\right),\underline{Y}:=g\left(Y\right)\\ \underline{m}:=\mathrm{Dec}\left(\underline{X},\underline{Y}\right)\\ \mathrm{output}:\;\underline{m},{\mathrm{Leak}}_{X}\left(X\right),{\mathrm{Leak}}_{Y}\left(Y\right) \end{array}\right\} \end{array}$$
3. In addition, of course, we can also consider a combination of the above, where tampering depends on the leakage, and the leakage is also part of the tampering output:
  $$\begin{array}{c} {\mathrm{TamperLeak}}_{m}^{f,g,{\mathrm{Leak}}_{X},{\mathrm{Leak}}_{Y}}=\left\{\begin{array}{c} (X,Y)\leftarrow \mathrm{Enc}(m),\\ \underline{X}:=f\left(X,{\mathrm{Leak}}_{Y}\left(Y\right)\right),\underline{Y}:=g\left(Y,{\mathrm{Leak}}_{X}\left(X\right)\right)\\ \underline{m}:=\mathrm{Dec}\left(\underline{X},\underline{Y}\right)\\ \mathrm{output}:\;\underline{m},{\mathrm{Leak}}_{X}\left(X\right),{\mathrm{Leak}}_{Y}\left(Y\right) \end{array}\right\} \end{array}$$

In all of the above, we expect the (modified) simulator to be indistinguishable from the tampering experiment. In the case of the experiment 2 and experiment 3, we slightly modify the simulator: The $D^{f,g,{\mathrm{Leak}}_{X},{\mathrm{Leak}}_{Y}}$ simulates not only the message but also the leakage:

$$\begin{array}{c} {\mathrm{TamperLeak}}_{m}^{f,g,{\mathrm{Leak}}_{X},{\mathrm{Leak}}_{Y}}\approx_{\varepsilon }\left\{\begin{array}{c} \left(d,\ell_{X},\ell_{Y}\right)\leftarrow D^{f,g,{\mathrm{Leak}}_{X},{\mathrm{Leak}}_{Y}}\\ ifd=\mathrm{same}\;\mathrm{then}\;\mathrm{output}\;m,\ell_{X},\ell_{Y}\\ \mathrm{otherwise}\;\mathrm{output}\;d,\ell_{X},\ell_{Y}. \end{array}\right\} \end{array}$$

**Remark** **1.** *Usually, we consider ${\mathrm{Leak}}_{X},{\mathrm{Leak}}_{Y}$ to be bounded output size leakages. In addition, ${\mathrm{Leak}}_{X}$ and ${\mathrm{Leak}}_{Y}$ might be a series of adaptive leakages depending on each other; then, one has to apply Lemma 4 to obtain the independence of X and Y given the leakages. We have to remark here that Lemma 4 states that X and Y are independent given ${\mathrm{Leak}}_{X}=\ell_{X}$ and ${\mathrm{Leak}}_{Y}=\ell_{Y}$; however, one has to remain vigilant since $X|{\mathrm{Leak}}_{X}=\ell_{X}$ and $Y|{\mathrm{Leak}}_{Y}=\ell_{Y}$ might not be efficiently sampleable sources; thus, the extension to the adaptive leakage case is straightforward only in the information theoretic world.*

**Remark** **2.** *The second definition might seem a bit artificial; however, it is quite useful for technical reasons. Sometimes, non-malleable code is merely one of many building blocks of bigger protocol/application, and the leakage is a byproduct of the technical proof—other parts of the protocol might behave differently depending on the non-malleable encoding (which is most conveniently modeled as a leakage), thus non-malleable code is tampered in a usual way while the rest of the protocol leaks extra information.*

The first compiler that returns a leakage resilient (with respect to the variant 1) non-malleable code was given by [20]; it could tolerate up to $\frac{1}{12}$ leakage rate (i.e., output size of leakage functions can be up to $\frac{1}{12}$ of the input size), but it required a symmetric decoder (Dec(X,Y)=Dec(Y,X)). Later, Ball, Guo, and Wichs gave a better compiler:

**Theorem** **3** (from [46,48])**.** *For any $\alpha \in [0,\frac{1}{4})$, there exists a compiler that takes any 2—split state non-malleable code and outputs a leakage resilient (with respect to definition variants 1, 2, and 3) non-malleable code with leakage rate α. The rate of the new code is Θ(original−rate), and the error stays the same except for an extra exp(−Ω(n)) factor (where n is the new code’s length).*

**Remark** **3** (from [46])**.** *Originally, it only showed that their compiler worked for the variant 1. However, Ref. [48] later extended their analysis to the latter variants.*

In addition, for the variants 2 and 3, we have the following result:

**Theorem** **4** (from [53]). *Any 2—split-state ε—non-malleable code is also $2^{t}\cdot \varepsilon —$non-malleable code that tolerates up to t bits of leakage (with respect to Definition 2 or 3).*

**Remark** **4.** *Originally, the above paper considered Definition 2 only, but simple inspection of the proof gives the security with respect to the variant 3 too.*

The idea of the proof is quite simple: we guess the leakage functions (thus the penalty $2^{t}$) and tampering function check if the leakage is consistent with their view; if any of the views does not match the guessed leakage, then the tampering aborts (*f* or *g* outputs ⊥, and the decoder aborts). Else, if the guessed leakage is consistent with the views of the tampering functions, the tampering happens as intended.; the above expands the power of tampering functions: instead of $f,g:{\left\{0,1\right\}}^{n}\to {\left\{0,1\right\}}^{n}$, we have $f,g:{\left\{0,1\right\}}^{n}\to {\left\{0,1\right\}}^{n}\cup \left\{⊥\right\}$; this is without a loss of generality—in a similar fashion as in Theorem 2, we can show that adding ⊥ as a possible output of the tampering functions does not break the definition.

Later, Ref. [35] expanded the result from Theorem 4 for augmented (see Section 3.4) non-malleable codes.

**Theorem** **5** (from [35])**.** *Any 2—split-state augmented ε—non-malleable code is also an augmented $2^{t}\cdot \varepsilon —$non-malleable code that tolerates up to t bits of leakage (with respect to Definition 2 or 3).*

Similarly, the [46] compiler also preserves the augmented property:

**Theorem** **6** (from [48])**.** *For any α∈[0,1/4), 2-split-state augmented ε—non-malleable code can be compiled into an augmented split-state ε+exp(−Ω(n))-non-malleable code with rate Θ(original−rate) and leakage rate α (with respect to any variant above).*

### 3.4. Augmented NMCS

Many applications require an extra property, namely that adversary on top of receiving a tampered message can get one of the states (similar to the leakage resilience discussed above).

**Definition** **8** (Left-augmented NMC)**.** *Let (Enc:M→X×X,Dec:X×X→M∪{⊥}) be an encoding scheme. For f,g:X→X and, for any m∈M, define the experiment ${\mathrm{Tamper}}_{m}^{f,g}$ as:*

$$\begin{array}{c} {\mathrm{Tamper}}_{m}^{f,g}=\left\{\begin{array}{c} (X,Y)\leftarrow \mathrm{Enc}(m),\\ \underline{X}:=f\left(X\right),\underline{Y}:=g\left(Y\right)\\ \underline{m}:=\mathrm{Dec}\left(\underline{X},\underline{Y}\right)\\ output:\underline{m},X \end{array}\right\} \end{array}$$

*We say that the encoding scheme (Enc,Dec) is* left-augmented ε-non-malleable in a 2−split-state model*if, for every function f,g:X→X, there exists a distribution $D^{f,g}$ on M∪{same,⊥} (without the access to the original message) such that, for every m∈M, we have*

$$\begin{array}{c} {\mathrm{Tamper}}_{m}^{f,g}\approx_{\varepsilon }\left\{\begin{array}{c} \left(d,x\right)\leftarrow D^{f,g}\\ ifd=same\;then\;output\;m,x\\ otherwise\;output\;d,x. \end{array}\right\} \end{array}$$

Symmetrically, we can consider the right-augmented property, where the right state is revealed.

Most of the known constructions like [9,29,30,31,35] are augmented (both left and right augmented). Interestingly [33], the non-malleable randomness encoder (see Section 6.1 for details) is right-augmented but not left-augmented.

### 3.5. Simulation vs. Game

Definition 7 is the most common simulation-based definition. However, in some situations, it is actually more convenient to consider a game based definition, where the adversary picks two messages $m_{0},m_{1}$; the challenger encodes $m_{b}$ for uniformly chosen *b*, and the adversary has to guess *b* based on the tampering of $\mathrm{Enc}\left(m_{b}\right)=\left(X_{b},Y_{b}\right)$.

In particular, the following alternative definition of a non-malleable code will give a smoother transition to the subsequent definitions in this section.

The transition from a simulator to a game is not quite trivial: let $\mathrm{Enc}(m_{0})=L,R$ and imagine the tampering with f(X)=L and g(Y)=R; now, both messages have been completely overwritten and both tampering experiments should output $m_{0}$. However, notice that tampering experiment ${\mathrm{Tamper}}_{m_{0}}^{f,g}$ has two options: it can answer $m_{0}$ or it can answer same, while ${\mathrm{Tamper}}_{m_{1}}^{f,g}$ can only answer $m_{0}$. In the above example, ${\mathrm{Tamper}}_{m_{0}}^{f,g}$ can not answer same else it will be distinguishable. To solve the dilemma, we have to add an extra “helper” sitting inside the tampering experiment that will decide if the tampering experiment should output same or $\underline{m}$.

**Definition** **9** (Game definition for non-malleable code, from [15]). *We say that an encoding scheme (Enc:M→X×X,Dec:$X\times X\to M\cup \left\{⊥\right\})$ is*ε-non-malleable in the split-state model*if, for every function f,g:X→X, there exists a family of distributions ${\left\{D_{x,y}^{f,g}\right\}}_{x,y\in X}$ each on {0,1} such that for every $m_{0},m_{1}\in M$*

$${\mathrm{Tamper}}_{m_{0}}^{f,g}\approx_{\varepsilon }{\mathrm{Tamper}}_{m_{1}}^{f,g}$$

*where*

$$\begin{array}{c} {\mathrm{Tamper}}_{m}^{f,g}=\left\{\begin{array}{c} (X,Y)\leftarrow \mathrm{Enc}(m),\\ output\;same\;if\mathrm{Dec}\left(X,Y\right)=\mathrm{Dec}\left(f\left(X\right),g\left(Y\right)\right)\wedge D_{X,Y}^{f,g}=0\\ else\;output:\mathrm{Dec}(f(X),g(Y)) \end{array}\right\} \end{array}$$

In [15] (Appendix A), the authors show the equivalence of Definitions 7 and 9.

**Theorem** **7** (from [15]). *If (Enc,Dec) is an ε—non-malleable code according to the game-based definition, then it is also an ε—non-malleable code according to the definition from [1].*

**Theorem** **8** (from [15]). *If (Enc,Dec) is an ε—-non-malleable code according to the definition from [1], then it is 4ε—non-malleable code according to the game-based definition.*

To prove the above, the authors construct explicit “helper” distribution. There was another game-based definition already considered in [1], but the above definition is easier to generalize to the definition for stronger notions of non-malleable codes.

### 3.6. Strong, Super, and Super-Strong Variants

Some results in the literature like [12,14] have considered a notion of super-strong non-malleable codes. We start with the following intermediate notion of super non-malleable codes introduced in [15]. In this variant, if the tampering is successful and non-trivial i.e., output is not ⊥ or same, then the tampering experiment outputs the whole tampered codeword. In other words, we require that, for valid tampering, either tampering does not change the message, or even the tampered codeword itself does not carry any information about the original message.

**Definition** **10** (Super non-malleable code). *We say that an encoding scheme (Enc:M→X×X,Dec:$X\times X\to M\cup \left\{⊥\right\})$ is* ε-super non-malleable in a split-state model*if, for every function, f,g:X→X, there exists a family of distributions ${\left\{D_{x,y}^{f,g}\right\}}_{x,y\in X}$ each on {0,1} such that for every $m_{0},m_{1}\in M$*

$${\mathrm{SuperTamper}}_{m_{0}}^{f,g}\approx_{\varepsilon }{\mathrm{SuperTamper}}_{m_{1}}^{f,g}$$

*where ${\mathrm{SuperTamper}}_{m}^{f,g}=$*

$$\begin{array}{c} \left\{\begin{array}{c} (X,Y)\leftarrow \mathrm{Enc}(m),\\ output\;same\;if\mathrm{Dec}\left(X,Y\right)=\mathrm{Dec}\left(f\left(X\right),g\left(Y\right)\right)\wedge D_{X,Y}^{f,g}=0\\ else\;if\mathrm{Dec}(f(X),g(Y))=⊥output⊥\\ else\;output:\left(f(X),g(Y)\right) \end{array}\right\} \end{array}$$

**Remark** **5.** *This definition is clearly stronger than the standard version, since, given the tampered codeword, we can apply the decoder and obtain the tampered message.*

In [1], the authors considered a strong variant. This is a variant that follows the standard definition closely except it puts a restriction on the use of same—it can only be outputted only if f(X)=X∧g(Y)=Y (Notice that outputting same in that case is unavoidable). This variant is perhaps the closest to the intuition; if the codeword is tampered with, then it is either invalid or it decodes to something independent of the original message.

**Definition** **11** (Strong non-malleable code). *We say that an encoding scheme (Enc:M→X×X,Dec:$X\times X\to M\cup \left\{⊥\right\})$ is* ε-strong non-malleable in the split-state model*if for every function f,g:X→X and for every $m_{0},m_{1}\in M$*

$${\mathrm{StrTamp}}_{m_{0}}^{f,g}\approx_{\varepsilon }{\mathrm{StrTamp}}_{m_{1}}^{f,g}$$

*where*

$$\begin{array}{c} {\mathrm{StrTamp}}_{m}^{f,g}=\left\{\begin{array}{c} (X,Y)\leftarrow \mathrm{Enc}(m),\\ outputsame\;if\;\left(X,Y)=(f(X),g(Y)\right)\\ else\;if\mathrm{Dec}(f(X),g(Y))=⊥output⊥\\ else\;output:\mathrm{Dec}(f(X),g(Y)) \end{array}\right\} \end{array}$$

Notice that above we do not need a “helper” distribution anymore since the conditions to output same are so restrictive.

Finally, one can consider both the super and strong version. Here, we require that same can only be outputted if f(X)=X∧g(Y)=Y, and, if the codeword is valid and not trivially tampered with, then the whole tampered codeword does not reveal any information about the original message.

**Definition** **12** (Super strong non-malleable code). *We say that an encoding scheme (Enc:M→X×X,Dec:$X\times X\to M\cup \left\{⊥\right\})$ is ε-super strong non-malleable in the split-state model if for every function f,g:X→X and for every $m_{0},m_{1}\in M$*

$${\mathrm{SupStrTamp}}_{m_{0}}^{f,g}\approx_{\varepsilon }{\mathrm{SupStrTamp}}_{m_{1}}^{f,g},$$

*where*

$$\begin{array}{c} {\mathrm{SupStrTamp}}_{m}^{f,g}=\left\{\begin{array}{c} (X,Y)\leftarrow \mathrm{Enc}(m),\\ output\;same\;if\left(X,Y)=(f(X),g(Y)\right)\\ else\;if\mathrm{Dec}(f(X),g(Y))=⊥output⊥\\ else\;output:\left(f(X),g(Y)\right) \end{array}\right\} \end{array}$$

Examples of the codes:

- Ref. [8] is not super and not strong;
- Ref. [9] is super but not strong;
- All non-malleable extractors including [29,30,31] are strong but not super;
- Ref. [9] compiled with [15] is super and strong.

**Informal** **Theorem** **1** (from [15])**.** *There exists a compiler that turns any super non-malleable code (with certain sampling properties which we discuss below) in a 2-split state model into a super-strong non-malleable code in a 2—split state model, at a minimal loss to the rate of the code. The above compiler also turns a non-malleable code into a strong non-malleable code.*

The idea behind the compiler is to introduce a certain level of circularity: $\mathrm{Enc}(m||{\mathrm{check}}_{X},$${\mathrm{check}}_{Y})=X,Y$; in other words, the codeword encodes its own “checks”. Notice that the difference between the strong and not-strong variant is only in the use of same output. The checks ensure that, if the code was tampered with and still decodes to the same message, then the checks remain unchanged—this leads to the decoder error since the checks will not match the changed codeword.

This approach has a problem: the circularity introduced above does not necessarily allow for efficient encoding, and thus there are additional requirements on the underlying non-malleable code. The authors show that the extra assumptions are fulfilled by the code from [9], thereby giving a super-strong non-malleable code.

### 3.7. Continuous Non-Malleable Codes

We can push the definition further; imagine that the codeword is tampered not once, but multiple times. This is the idea behind continuous non-malleable codes. Meanwhile, in principle, we can take any of the four variants: standard, strong, super, super-strong, and extend the definition to multiple tamperings for various technical reasons (The main problem of non-super variants is that, immediately after the first tampering, X,Y are not independent anymore given $\mathrm{Dec}(f_{1}\left(X\right),g_{1}\left(Y\right))$; this causes huge technical problems; thus, in practice, it is actually easier to aim for the strongest variant. Intuitively speaking X,Y remain “somewhat-independent” given f(X),g(Y), where by “somewhat-independent”, we mean that X,Y still form a valid codeword, but revealing extra information does not add any additional correlations) the super-strong extension is the one that received attention.

There are again four variants that stem from two possible flags: self-destruct (yes/no) and persistence (yes/no).

*Self-destruct* decides what happens when one of the tamperings outputs ⊥—should we stop the experiment, or should we allow the adversary to continue tampering? We will discuss later that non-self-destruct codes do not exist for the most of the reasonable tampering families.

*Persistence* (often referred to as resettability) decides how the tampering is applied; say codeword *c* was tampered into $c^{\prime }$, is the next tampering applied to original *c*, or should it be applied on top of $c^{\prime }$? As long as $c\to c^{\prime }$ is a bijection, that is not a problem, but, if the tampering function was very lossy given $c^{\prime }$, we can not recreate *c*, thus this becomes a non-trivial choice. Indeed, later we will discuss impossibility results that strongly separate persistent (not-resettable) and non-persistent (resettable) codes.

**Remark** **6** (Note on two-source non-malleable extractors)**.** *It is important to stress few things: two-source non-malleable extractors do not output *⊥*, thus (for the same reason why non-self-destruct codes do not exist for reasonable tampering classes) we can not consider a continuous version of them. However, we can, and usually do, consider a t—times tampering variants, where a two-source non-malleable extractor is tampered t times for some fixed in advance t.*

**Definition** **13** (Continuous Non-Malleable Code). *Ref. [14] define four types of continuous non-malleable codes based on two flags: sd∈{0,1}*(self-destruct)* and prs∈{0,1}* (persistent)*. We say that an encoding scheme (Enc:M→X×X,Dec:$X\times X\to M\cup \left\{⊥\right\})$ is*(T,ε)-continuous [sd,prs] non-malleable in the split-state model*if, for every Adversary A and for every $m_{0},m_{1}\in M$*

$${\mathrm{ConTamper}}_{A,T,m_{0}}\approx_{\varepsilon }{\mathrm{ConTamper}}_{A,T,m_{1}},$$

*where ${\mathrm{ConTamper}}_{A,T,m}=$*

$$\begin{array}{c} \left\{\begin{array}{c} (X,Y)\leftarrow \mathrm{Enc}(m),\\ f_{0},g_{0}\equiv \mathrm{id},\\ \mathrm{Repeat}\;i=1,2,...,T\\ \;A\;chooses\;functions\;f_{i}^{\prime },g_{i}^{\prime }\\ \;\mathrm{if}\mathrm{prs}=1\mathrm{then}f_{i}=f_{i}^{\prime }\circ f_{i-1},\;g_{i}=g_{i}^{\prime }\circ g_{i-1}\\ \;\;\mathrm{else}f_{i}=f_{i}^{\prime },\;g_{i}=g_{i}^{\prime }\\ \;\mathrm{if}(f_{i}\left(X\right),g_{i}\left(Y\right))=\left(X,Y\right)\mathrm{then}\mathrm{output}\mathrm{same}\\ \;\;\mathrm{else}\\ \;\;\mathrm{if}\mathrm{Dec}(f_{i}\left(X\right),g_{i}\left(Y\right))=⊥\mathrm{then}\mathrm{output}⊥\mathrm{if}\mathrm{sd}=1\mathrm{then}\mathrm{experiment}\mathrm{stops}\\ \;\;\;\mathrm{else}\mathrm{output}(f_{i}\left(X\right),g_{i}\left(Y\right))\mathrm{if}\mathrm{prs}=1\mathrm{then}\mathrm{experiment}\mathrm{stops} \end{array}\right\} \end{array}$$

**Remark** **7.** *In the case of persistent tampering, the above definition by [14] assumes that the tampering experiment stops if there is a non-trivial tampering that does not decode to ⊥, since, in this case, the adversary learns the entire tampered codeword and can simulate the remaining tampering experiment himself (since the tampering is persistent).*

**Remark** **8.** *In any model allowing bitwise tampering, in particular the 2—split state model, it is not difficult to conclude that the* non-self-destruct * property is impossible to achieve even in the case of persistent tampering if the space of messages contains at least three elements. To see this, notice that one can tamper the codeword $c=(c_{1},c_{2},c_{3},\ldots )$ to obtain $c_{1}^{\prime }=\left(0,c_{2},\ldots \right)$. The adversary then obtains the output of the tampering experiment, which is same if and only if $c_{1}=0$. Thus, the adversary learns $c_{1}^{☆}=c_{1}$ and continues the tampering experiment with $\left(c_{1}^{☆},0,c_{3},\ldots \right)$ (note that this tampering is persistent). Thus, the adversary can continue to learn the codeword one bit at a time, thereby learning the entire codeword in N steps where N is the length of the codeword.*

The constructions:

**Theorem** **9** (from [15]). *If (Enc,Dec) is an ε-super strong non-malleable code in the 2—split-state model, then (Enc,Dec) is a (T,(2T+1)ε)—continuous self-destruct, persistent non-malleable code in the 2—split-state model. This combined with [9] compiled with [15] gives an explicit and efficient continuous self-destruct persistent non-malleable code in the 2—split-state model.*

**Remark** **9.** *The number of tampering rounds T does not have to be specified in advance (unlike with two-source non-malleable extractors). We expect the number of tamperings to be polynomial, and ε to be negligible; one can plug those in and obtain a code with unlimited (but polynomial) number of tamperings and security $\varepsilon^{\alpha }$ for any α<1.*

The idea behind the theorem is as follows: there are only two output patters that we can observe: either there will be some number of same outputs followed by a ⊥ or followed by the tampered codeword $c^{\prime }$. The authors argue that the long same chain does not teach us much, thus the only tampering that really matters is the last one (the one that leads to not-same). Thus, the continuous tampering is actually reduced to the one non-trivial tampering; we just have to pay a small price in epsilon, since we basically have to guess in which round the non-same tampering will happen.

**Remark** **10.** *The above technique was extended and generalized by [54] for the other tampering classes. In particular, the authors achieve continuous NMC against persistent decision tree tampering.*

**Informal** **Theorem** **2** (from [16])**.** *There exists an explicit and efficient self-destruct, non-persistent (resettable) continuous non-malleable code in the 8-split state model (i.e., where we have eight states instead of 2).*

**Remark** **11.** *Ref. [12] shows that non-persistent continuous non-malleable codes are impossible to construct in a 2-split state model. We know that eight states is enough, and we hypothesize that the idea behind [16] could be extended to give an existential (not efficient or explicit) 6-state construction. The exact number of states required to construct non-persistent code remains an opened question even in the non-explicit case.*

## 4. The Non-Malleable Code Construction via Inner Product

In this section, we show a construction of non-malleable codes via an inner product due to [9,49,50].

**Theorem** **10.** *There exist absolute constants $c,c^{\prime }>0$ such that the following holds. For any finite field $F_{p}$ of prime order, and any $n>c^{\prime }\log^{4}p$,*

$$(S_{n,p}^{2}\Rightarrow {\mathrm{AFF}}_{p},2^{-cn^{1/4}})\;.$$

We will prove the following theorem which immediately implies Theorem 10. To see this, consider the encoding function that takes as input an element of $x\in F_{p}$ and chooses uniformly random $L,R\in F_{p}^{n}$ conditioned on 〈L,R〉=x, and the decoding function Dec(ℓ,r) is defined as Dec(ℓ,r):=〈ℓ,r〉.

**Theorem** **11.** *There exist absolute constants $c,c^{\prime }>0$ such that the following holds. For any finite field $F_{p}$ of prime order, and any $n>c^{\prime }\log^{4}p$, let L,R be random variables uniform and independent in $F_{p}^{n}$, and $f,g:F_{p}^{n}\to F_{p}^{n}$ be arbitrary functions. Then,*

$$\Delta (\langle L,R\rangle ,\langle f\left(L\right),g\left(R\right)\rangle \;;\;U_{F_{p}},D\left(U_{F_{p}}\right)\;,$$

*where $U_{F_{p}}$ is uniform and independent of L,R, and D is a distribution over ${\mathrm{AFF}}_{p}$.*

We need the following result that can be seen as a generalization of the linearity test from [55] and that is discussed and proved in [9].

**Theorem** **12.** *Let p be a prime, and n be a positive integer. For any ε=ε(n,p)>0, $\gamma_{1}=\gamma_{1}\left(n,p\right)\leq 1$, $\gamma_{2}=\gamma_{2}\left(n,p\right)\geq 1$, the following is true. For any function $f:F^{n}\to F^{n}$, let $A\subseteq \left\{\left(x,f\left(x\right)\right)\;:\;x\in F^{n}\right\}\subseteq F^{2n}$. If $\left|A\right|\geq \gamma_{1}\cdot \left|F^{n}\right|$ and there exists some set B such that $\left|B\right|\leq \gamma_{2}\cdot p^{n}$, and*

$${\mathrm{Pr}}_{a,a^{\prime }\in A}\left[a---a^{\prime }\in B\right]\geq \varepsilon ,$$

*then there exists a linear map $M:F^{n}\to F^{n}$ such that*

$$\underset{(x,f(x))\in A}{\mathrm{Pr}}\left[f\left(x\right)=Mx\right]\geq p^{-O(\log^{6}\left(\frac{\gamma_{2}}{\gamma_{1}\varepsilon }\right))}\;.$$

### 4.1. Proof Sketch

Now we sketch the proof of Theorem 11 The following lemma shows that, for any large enough subdomain of $F^{n}\times F^{n}$ for which 〈L,R〉,〈f(L),g(R)〉 is not close to the desired distribution for some *D*, there exists a large enough subdomain on which *f* is linear.

**Lemma** **11.** *Let p be a prime, n a positive integer, and 0<t<n. Let $L\subseteq F_{p}^{n}$ such that $\left|L\right|\geq p^{n-t}$. Let L,R be independent random variables uniformly distributed in L and $F_{p}^{n}$, respectively. Then, either there exists a distribution G over ${\mathrm{AFF}}_{p}$ such that*

$$\Delta (\langle L,R\rangle ,\langle f\left(L\right),g\left(R\right)\rangle \;;\;U_{F_{p}},D\left(U_{F_{p}}\right)\leq p^{-t}\;,$$

*or there exists a subset $|L^{\prime }\subseteq L|$, a linear map $M\in {\left(F_{p}^{n}\right)}^{F_{p}^{n}}$, and a constant C such that $|L^{\prime }|\geq |L|\cdot p^{-Ct^{4}\log^{4}p}$, and f(x)=Mx for all $x\in L^{\prime }$.*

**Proof.**  We assume that

$$\Delta (\langle L,R\rangle ,\langle f\left(L\right),g\left(R\right)\rangle \;;\;U_{F_{p}},D\left(U_{F_{p}}\right)>p^{-t}\;,$$

as otherwise the result trivially holds. Then, by Lemma 7, there exist a∈F such that $\Delta \left(\langle L,R\rangle +a\langle f\left(L\right),g\left(R\right)\rangle \;;\;U_{F_{p}}\right)\geq p^{-t-2}$. Define functions $F,G:F^{n}\to F^{2n}$ as follows:

F(x)=(x,f(x)),G(y)=(y,ag(y)).

We have that $\Delta \left(\langle F\left(L\right),G\left(R\right)\rangle \;;\;U_{F_{p}}\right)\geq p^{-t-2}$. Applying Lemma 8, we get that, for $\left(L^{\prime },R^{\prime }\right)$ i.i.d to (L,R), we have

$$\mathrm{Pr}\left[\langle F\left(L^{\prime }\right),G\left(R^{\prime }\right)\rangle =\langle F\left(L\right),G\left(R\right)\rangle \right]\geq \frac{1}{p}+\frac{1}{p^{2t+5}}.$$

Applying Lemma 9 with $X=F\left(L\right),Y=G\left(R\right),X^{\prime }=F\left(L^{\prime }\right),Y^{\prime }=G\left(R^{\prime }\right)$, we get that

$$\mathrm{Pr}\left[\langle F\left(L\right)-F\left(L^{\prime }\right),G\left(R\right)\rangle =0\right]\geq \frac{1}{p}+\frac{1}{p^{2t+5}}.$$

Define

$$B:=\left\{\alpha \in F_{p}^{2n}:\mathrm{Pr}\left[\langle \alpha ,G\left(R\right)\rangle =0\right]\geq \frac{1}{p}+\frac{1}{p^{2t+6}}\right\}.$$

Let B∈B be uniform. Then, $\Delta \left(\langle B,G\left(R\right)\rangle ,U_{F_{p}}\right)\geq \frac{1}{p^{2t+6}}$. In addition, since *R* is uniform in $F_{p}^{n}$, G(R) has min-entropy nlogp. Hence, by Lemma 2, we have $H_{\infty }\left(B\right)\leq \left(n+4t+13\right)\cdot \log p$, which implies $\left|B\right|\leq p^{n+4t+13}$. Furthermore, we have that

$$\mathrm{Pr}\left[\langle F\left(L^{\prime }\right)-F\left(L^{\prime \prime }\right),G\left(R^{\prime }\right)\rangle =0\right]\leq \mathrm{Pr}\left[F\left(L^{\prime }\right)-F\left(L^{\prime \prime }\right)\in B\right]+\frac{1}{p}+\frac{1}{p^{2t+6}}.$$

Thus, we must have that

$$\mathrm{Pr}\left[F\left(L^{\prime }\right)-F\left(L^{\prime \prime }\right)\in B\right]\geq \frac{1}{p^{2t+5}}-\frac{1}{p^{2t+6}}\geq \frac{1}{p^{2t+6}}.$$

Thus, using Theorem 12, we obtain that there exists a linear map $M:F^{n}\to F^{n}$ for which

$$\underset{x\in F^{n}}{\mathrm{Pr}}\left[Mx=f\left(x\right)\right]\geq p^{-O(t^{4}\log^{4}p)}\;.$$

□

We now show that, if *f* is linear, then we get the desired distribution.

**Lemma** **12.** *Let p be a prime, n a positive integer, and 0<s<n. Let $L^{\prime }\subseteq F_{p}^{n}$ such that $|L^{\prime }|\geq p^{n-s}$, and f(x)=Mx for all $x\in F_{p}^{n}$, where M is a linear map in ${\left(F_{p}^{n}\right)}^{F_{p}^{n}}$. Let L,R be independent random variables uniformly distributed in $L^{\prime }$ and $F_{p}^{n}$, respectively. Then, there exists a distribution G over ${\mathrm{AFF}}_{p}$ such that*

$$\Delta (\langle L,R\rangle ,\langle f\left(L\right),g\left(R\right)\rangle \;;\;U_{F_{p}},G\left(U_{F_{p}}\right)\leq p^{-s}\;.$$

**Proof.**  We will prove that the given statistical distance is small for almost all fixing of $R\in F_{p}^{n}$, and hence concludes the desired result.

Let S be the set of all $x\in F_{p}^{n}$ such that $\Delta \left(\langle L,s\rangle ,U_{F_{p}}\right)>p^{-3-s}$. By Lemma 2, $\left|S\right|\leq p^{3s+8}$.

Note that

$$\langle f\left(L\right),g\left(r\right)\rangle =\langle ML,g\left(r\right)\rangle =\langle L,M^{T}g\left(r\right)\rangle \;.$$

Thus, without loss of generality, we assume *M* to be the identity function, and replace *g* by $M^{T}g$. Assume (〈L,r〉,〈f(L),g(r)〉) is not $p^{-s-1}$-close to $U_{F_{p}},G\left(U_{F_{p}}\right)$ for any *G* distributed over ${\mathrm{AFF}}_{p}$. This means that, for any $a\in F_{p}$, (〈L,r〉,〈f(L),g(r)〉) is not $p^{-s-1}$-close to $U_{F_{p}},aU_{F_{p}}+B$, for some random variable *B* independent of $U_{F_{p}}$. By Lemma 7, for every $a\in F_{p}$, there exists $b\in F_{p}$ such that

(2) $$\Delta \left(\langle L,r+b\left(g\left(r\right)-ar\right)\rangle \;;\;U_{F_{p}}\right)\geq p^{-3-s}\;.$$

We will show that this implies that $r\in F_{p}S+F_{p}S$.

$$r\in \{\alpha_{1}x_{1}+\alpha_{2}x_{2}\;|\;\alpha_{1},\alpha_{2}\in F_{p},x_{1},x_{2}\in S\}\;.$$

By Equation (2) with a=0, there is some $b=b^{*}$ such that $r+b^{*}g\left(r\right)=x_{1}\in S$. We assume without loss of generality that $b^{*}\neq 0$ since, if $b^{*}=0$, then r∈S, and the desired statement is true.

Letting $a=-1/b^{*}$, we have that there exists *b* such that $r\left(1+b/b^{*}\right)+bg\left(r\right)=x_{2}\in S$.

Combining, we get that $r=\left(bx_{1}---bx_{2}\right)/b^{*}$, thereby proving that $r\in F_{p}S+F_{p}S$. Thus, by Lemma 6, the desired statistical distance is at most

$$\frac{p^{6s+20}}{p^{n}}\cdot 1+p^{-1-s}\leq p^{-s}\;.$$

□

#### Finishing the Proof Sketch

By Lemma 11, whenever 〈L,R〉,〈f(L),g(R)〉 are not close to U,D(U) for some affine function *D*, we can always find a large subset of the domain on which *f* is linear, and thus, using Lemma 12, we obtain that, on this subset, 〈L,R〉,〈f(L),g(R)〉 is close to U,D(U) for some *D*. We thus continue to find a large subset of the domain on which 〈L,R〉,〈f(L),g(R)〉 is close to U,D(U) until we are left with a very small fraction of the entire domain. The result then follows from Lemma 6.

### 4.2. Non-Malleable Codes against Affine Tampering in $F_{p}$

To complete the construction of non-malleable codes in the split-state model, we need non-malleable codes against affine tampering in $F_{p}$. The following was shown in [49].

**Theorem** **13.** *For any integer k>0, and $p>2^{4k}$,*

$$({\mathrm{AFF}}_{p}\Rightarrow {\mathrm{NM}}_{k},2^{-\Omega (k)})\;.$$

The construction for this is quite simple. A so-called affine-evasive subset *S* of $F_{p}$ of size significantly larger than $2^{k}$ was constructed with the property that for any fixed $\left(a,b\right)\in F_{p}\times F_{p}\backslash \left\{1,0\right\}$, we have that |aS+b∩S|≪|S|. The set *S* is then partitioned into $K=2^{k}$ subsets $S_{1},\ldots ,S_{K}$, and the encoding of the *i*-th message is a uniformly random element of $S_{i}$, and all elements in $F_{p}$ not in *S* decode to a special symbol ⊥.

## 5. The Non-Malleable Codes via Two-Source Non-Malleable Extractors

Towards the goal of constructing non-malleable codes, Cheraghchi and Guruswami [27] introduced non-malleable extractors as a stronger primitive that immediately yields efficient non-malleable codes as long as the preimage of the extractor is efficiently sampleable. Informally, a non-malleable two-source extractor nmExt guarantees that, for any independent random sources X,Y, and any functions f,g with at least one of them having no fixed points, nmExt(X,Y) is indistinguishable from uniform even given nmExt(f(X),g(Y)) (We say that the extractor is a strong non-malleable two-source extractor if, for any independent random sources X,Y, and any functions f,g with at least one of them having no fixed points, nmExt(X,Y) is indistinguishable from uniform even given nmExt(f(X),g(Y)) and *Y*). It is easy to see that a non-malleable two-source extractor gives non-malleability for a uniformly random message (average-case security) while a non-malleable code achieves non-malleability for every message (worst-case security). A non-malleable two-source extractor can be transformed into a non-malleable code (Enc,Dec) by setting $\mathrm{Enc}\left(m\right):={\mathrm{nmExt}}^{-1}\left(m\right)$, and Dec(x,y):=nmExt(x,y).

***NME to NMC: Limitations.*** We note that the transformation from nm2Ext to NMCs requires arguing worst-case security from average-case security, which incurs a factor $2^{\left|\mathrm{message}\;\mathrm{size}\right|}$ penalty in the security parameter. Most results on building 2-split-state NMCs have focused on improving the rate of non-malleable two-source extractors and relied on this *lossy* transformation to build NMCs.

Since their conception, the non-malleable two-source extractors went a long way and found independent applications, from the network extraction [56], to variants of privacy amplification [57]. More importantly, we know numerous connections and reductions between two-source extractors, seeded non-malleable extractors, and two-source non-malleable extractors (see [29,30,57,58]). This gives us hope that further progress in the constructions of these objects might give us an explicit two-source extractor with a negligible error and a low entropy requirements for both sources.

## 6. Rate Amplification Techniques

Another useful technique towards improving the rate of NMC constructions is *rate amplification* or *bootstrapping*. It is a recurring theme in cryptography to combine a scheme with a very strong security but bad efficiency with a scheme with bad security but a great efficiency in such a way that the resulting scheme inherits the best of both worlds: good security and efficiency.

In the context of non-malleable codes, it was first used by [13]. The authors achieved a rate 1 non-malleable code against bitwise tampering and permutations by combining the rate 0 scheme (from [59]) with an error correcting a secret sharing scheme (that has no non-malleability guarantee). In the context of a 2-split state tampering, it was used by [32,33,34,35].

The abstract idea is to use an efficient code to encode the message, while the bad rate code will encode tags and checks independent of the message’s size. What is left is to argue that those tags will guarantee the security of the construction.

In the remainder of this section, we will dive deeper into the construction of [32,33,35]. The latter paper achieves a current state-of-the-art rate of $\frac{1}{3}$, but it strongly builds on the construction of the former paper; thus, we can not discuss one without the other.

### 6.1. Technical Overview of “Non-Malleable Randomness Encoders and Their Applications”

This paper ([33]) does not build non-malleable code, but it forms a crucial building block for the construction of [35].

**Informal** **Theorem** **3** (Main Result of [33])**.** *There exists an efficient, information theoretically secure non-malleable randomness encoder with a rate arbitrarily close to $\frac{1}{2}$, and the negligible error.*

Kanukurthi, Obbattu, and Sekar [33] introduced the notion of *non-malleable randomness encoders* (NMRE). Similar to a 2-split-state NMC, a 2-split-state NMRE consists of two independently tamperable states *L* and *R*. Contrary to an NMC, where the encoder encodes arbitrary messages, an NMRE’s encoder outputs *L* and *R* such that they decode to a random string, and herein lies all the difference: as we have already discussed in the context of non-malleable extractors, it might not be possible to efficiently find the preimage of the specific message, or the security parameter might be too small to allow for fixing the specific message in a blackbox way.

**Figure 1:**
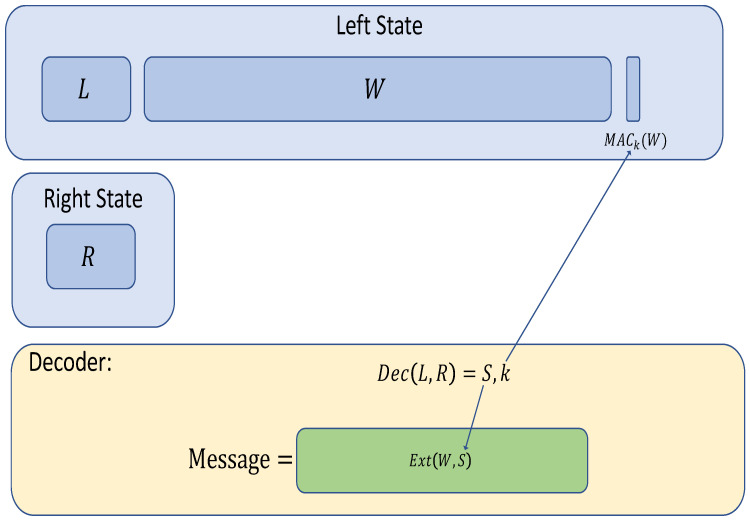
Construction of the non-malleable randomness encoder by [33].

While the problem of building high-rate NMCs has eluded researchers for over a decade, we know how to build NMREs with rate $\frac{1}{2}$ (see [33]). At the same time, we emphasize that obtaining a high-rate NMC (instead of an NMRE) is critical for many applications (such as non-malleable commitments.) Informally, the NMRE of [33] (see Figure 1) picks the source *w* and seed *s* to a strong seeded extractor (Ext) as well as a key *k* to a message authentication code (MAC). The code consists of two states, left: $\ell ∥w∥\sigma_{w}$, and right: *r*, where ℓ,r are an encoding of s,k with any low-rate augmented non-malleable code, and $\sigma_{w}$ is a tag evaluated on *w* with *k* as the key. The codeword, if valid, decodes to Ext(w;s).

To denote the tampering of a variable *x*, we will use the $\underline{x}$ notation. The security proof can be split into an analysis of the following three cases:

- $P_{id}=\left\{\left(\ell ,w,\sigma_{w},r\right):\left(w,\sigma_{w}\right)=\left(\underline{w},\underline{\sigma_{w}}\right)\wedge \mathrm{NMDec}\left(\ell ,r\right)=\mathrm{NMDec}\left(\underline{\ell },\underline{r}\right)\right\}$. This partition corresponds to the adversary not tampering with the codeword. In this case, the codeword will decode to the same message.
- $P_{tag}=\left\{\left(\ell ,w,\sigma_{w},r\right):\left(w,\sigma_{w}\right)\neq \left(\underline{w},\underline{\sigma_{w}}\right)\wedge \mathrm{NMDec}\left(\ell ,r\right)=\mathrm{NMDec}\left(\underline{\ell },\underline{r}\right)\right\}$. This partition corresponds to the case where $s=\underline{s}$ and $k=\underline{k}$. Since the MAC’s key remains secure and hidden from the adversary, the codeword will decode to ⊥ with high probability via the security of the message authentication codes.
- $P_{rest}=\left\{\left(\ell ,w,\sigma_{w},r\right):\mathrm{NMDec}\left(\ell ,r\right)\neq \mathrm{NMDec}\left(\underline{\ell },\underline{r}\right)\right\}$. Finally, this partition represents the case when adversary did apply non-trivial tampering to ℓ,r. By the properties of non-malleable code, if the codeword falls into this partition (and the likelihood of falling into this partition is not too small) $\underline{S}$ is independent of *S* (even given *L*).
  Now we will proceed with the following trick: we will reveal $\underline{S}$ and *L*, since $\underline{W}$ is now a function of *W* only (We can ignore tag $\sigma_{w}$ as a tiny leakage, alternatively the tag can be moved inside the non-malleable encoding) we obtain that $H_{\infty }\left(W|\mathrm{Ext}\left(\underline{W},\underline{S}\right),\underline{S},L\right)\geq \left|W|-|\mathrm{Ext}|-\right|\underline{S}|$, where $|\underline{S}|$ penalty comes since $\underline{L}$ might have depended on *W* and thus $\underline{S}$ might depend on *W*.
  This is a spot where we need augmented property as *S* remains uniform and independent of *W*, $\underline{S}$ and *L*. Thus, as long as $H_{\infty }\left(W|\mathrm{Ext}\left(\underline{W},\underline{S}\right),\underline{S},L\right)>\left|\mathrm{Ext}\right|$, we will obtain that Ext(W,S) is uniform given $\mathrm{Ext}(\underline{W},\underline{S})$. This means that the original message remains uniform given the message after tampering. The only thing to ensure is that $\left|W|-|\mathrm{Ext}|-\right|\underline{S}\left|>|\mathrm{Ext}\right|$. Since the size of *S* is small, we roughly obtain that |W|>2|Ext|, which leads to the rate $\frac{1}{2}$.

In order to extend this construction to encode an arbitrary message *m*, one option would be to reverse sample *w* and *s* such that Ext(w;s)=m. Unfortunately, this will not work because, on the one hand, we require the seed *s* to be short (as it is encoded using a poor-rate NMC) and, on the other hand, given a source *w*, there will be at most $2^{\left|s\right|}$ possible messages that could have been encoded; thus, adversary tampering with *w* will likely be able to distinguish between two messages of his choice (since, only for one of them, there will exist $s_{i}$ such that $\mathrm{Ext}\left(w,s_{i}\right)=m_{i}$). In other words, to obtain any meaningful security, *s* needs to be as long as the message. However, if *s* is long, the above approach will not yield an improvement in the rate.

### 6.2. Technical Overview of “Rate One-Third Non-Malleable Codes”

**Theorem** **14** (Main Result of [35]). *There exists an efficient, information-theoretically secure ε-right-augmented (The right-augmented property guarantees that the right state of the NMC is simulatable independent of the message, along with the tampered message) non-malleable code in the 2-split-state model with rate 1/3. The authors give two instantiations of the scheme: the first gives a strikingly simple construction and achieves an error of 2${}^{-\Omega (\kappa^{1/5}/\mathrm{polylog}\left(\kappa \right))}$; the second instantiation loses out on the simplicity but achieves an error of ε = 2${}^{-\Omega (\frac{\kappa }{\log^{3}\kappa })}$, where κ is the size of the message.*

**Figure 2:**
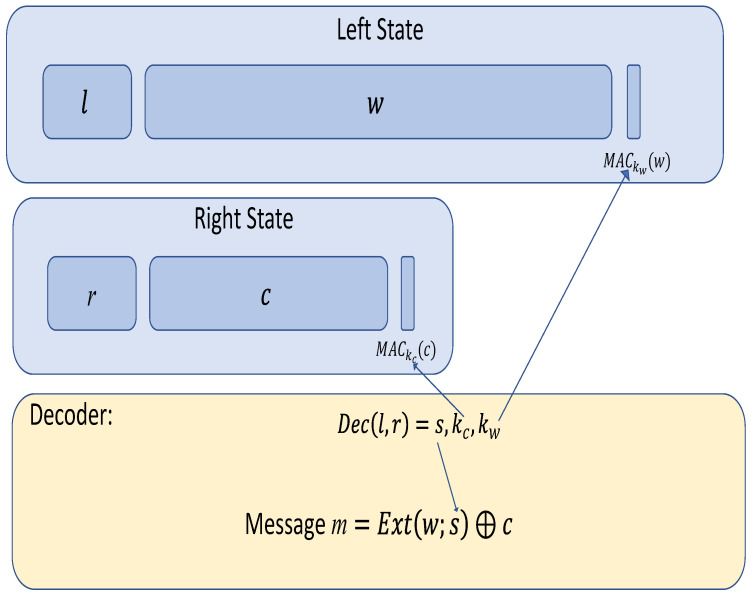
Overview of the construction from [35]. Blocks ℓ,r come from augmented non-malleable code. The encoder proceeds in steps: first, we randomly sample $s,k_{w},k_{c},w$ (all independently of the message we are encoding); then, we encode $s,k_{w},k_{c}$ using NMC into ℓ,r. We then set c=Ext(w;s)⊕m, and evaluate $\sigma_{c}$ as an MAC tag of *c* on key $k_{c}$, and $\sigma_{w}$ as an MAC tag of *w* on key $k_{w}$.

As we discussed earlier, fixing a specific message in the scheme of [33] is not possible. The idea is to add extra information to the right state that will allow for fixing a specific message. The construction described in Figure 2 goes as follows: as before, we will pick random w,s; then, we will fix c=Ext(w,s)⊕m, and, after that, we will pick two random keys $k_{c},k_{w}$ and encode using a non malleable code: $\mathrm{Enc}(s,k_{c},k_{w})=\ell ,r$. Finally, we calculate $\sigma_{w}$ an MAC of *w* under key $k_{w}$ and $\sigma_{c}$ a tag of *c* under key $k_{c}$. The encoding is left state: $\left(\ell ||w||\sigma_{w}\right)$ and right state: $\left(r||c||\sigma_{c}\right)$.

As a side note, we mention that the encoding scheme is identical to that due to Kanukurthi, Obbattu, and Sekar [32]. While [32] gave a four-state construction, Ref. [35] merged states to obtain a two-state construction.

We now offer an overview of the proof.

This construction uses the following building blocks: a message authentication code, a strong seeded extractor, and a low-rate non-malleable code which we shall use to encode the keys of the message authentication code and the seed for the seeded extractor. In addition, for a variable *X*, $\underline{X}$ will denote its tampering. We proceed with a slightly simplified sketch of the proof.

Proof Overview

The proof proceeds by partitioning the codeword space. We describe the partitions below:

- $P_{1}=\left\{\left(\ell ,w,\sigma_{w},r,c,\sigma_{c}\right):\left(w,\sigma_{w},c,\sigma_{c}\right)=\left(\underline{w},\underline{\sigma_{w}},\underline{c},\underline{\sigma_{c}}\right)\wedge \mathrm{NMDec}\left(\ell ,r\right)=\mathrm{NMDec}\left(\underline{\ell },\underline{r}\right)\right\}$. This partition captures the scenario when, even after tampering, the inner codeword (ℓ,r) decodes to the same message, and $W,\sigma_{w},C,\sigma_{c}$ remain unchanged; in this case, the final codeword must decode to the same message.
- $P_{2}=\left\{\left(\ell ,w,\sigma_{w},r,c,\sigma_{c}\right):\left(w,\sigma_{w},c,\sigma_{c}\right)\neq \left(\underline{w},\underline{\sigma_{w}},\underline{c},\underline{\sigma_{c}}\right)\wedge \mathrm{NMDec}\left(\ell ,r\right)=\mathrm{NMDec}\left(\underline{\ell },\underline{r}\right)\right\}$. $P_{2}$ captures the scenario when the decoding of the inner code remains unchanged after tampering, while one of the pairs $\left(W,\sigma_{w}\right)$ or $\left(C,\sigma_{c}\right)$ are changed; if this event occurs, then, using the security of MACs, the tampering is detected with overwhelming probability.
- $P_{3}=\left\{\left(\ell ,w,\sigma_{w},r,c,\sigma_{c}\right):\mathrm{NMDec}\left(\ell ,r\right)\neq \mathrm{NMDec}\left(\underline{\ell },\underline{r}\right)\right\}$. $P_{3}$ captures the scenario that the inner code is non-trivially tampered and does not decode to NMDec(L,R). The authors show that the tampered codeword is independent of the original message *m*. This is the most interesting case.

In order to prove non-malleability, we need to demonstrate the existence of a simulator whose outputs are indistinguishable from the output of the tampering experiment. The simulator does not use the message; however, it outputs a special symbol same to indicate that the tampered message is unchanged. The simulator’s output is run through a special wrapper function (typically called “Copy” function) that, in this case, outputs the original message.

The simulator generates the codeword $\left(\left(L,W,\sigma_{w}\right),\left(R,\tilde{C},{\tilde{\sigma }}_{c}\right)\right)$ of a random message. If this simulated codeword is in $P_{1}$, it outputs same. Recall that the wrapper function will then output the original message. If the simulated codeword is in $P_{2}$, the simulator outputs ⊥, else the simulator outputs $\mathrm{Dec}\left(\left(L,W,\sigma_{w}\right),\left(R,\tilde{C},{\tilde{\sigma }}_{c}\right)\right)$. (Note that the code is right-augmented i.e., it satisfies a stronger notion of security where the right state of the codeword can be revealed without breaking non-malleability.)

To prove non-malleability, we need to show that this behavior of the simulator is indistinguishable from that of the tampering experiment. To do this, we first need to argue that the probability of a codeword being in any given partition is independent of the message. The authors do it by showing how to determine a partition given small leakages from the left and right state and then arguing that those small leakages can not leak the encoded message, and thus the probability of falling into each partition can not depend on the encoded message (This proof relies on the secret sharing property of the non-malleable code as well as the security of the strong randomness extractor).

Next, the authors show that the output of the tampering experiment is, in each case, indistinguishable from the simulator’s output.

For the case where the codeword is in partition $P_{1}$, it is clear that the simulator output is identical to that of the tampering experiment. We, therefore, focus on the other two cases.

#### 6.2.1. Codeword is in $P_{2}$ i.e., $\mathrm{NMDec}\left(\underline{L},\underline{R}\right)=\mathrm{NMDec}\left(L,R\right)$

Intuitively, we would like to argue that the tag keys $K_{w},K_{c}$ will remain securely hidden from the adversary, and, if he decides to tamper with *W* or *C*, he will not be able to fake tags $\sigma_{w},\sigma_{c}$. Thus, either the whole codeword remains untampered (in which case, we are in $P_{1}$) or the new codeword will not be valid.

The standard approach would be to argue that, if $\mathrm{Pr}(\mathrm{NMDec}\left(\underline{L},\underline{R}\right)=\mathrm{NMDec}\left(L,R\right))$ is not too small, then

$$\begin{array}{c} \mathrm{Pr}(\mathrm{tampered}\;\mathrm{codeword}\;\mathrm{is}\;\mathrm{valid}\;\wedge \left(\underline{W},\underline{C}\right)\neq \left(W,C\right)\;|\;\mathrm{NMDec}\left(\underline{L},\underline{R}\right)=\mathrm{NMDec}\left(L,R\right)) \end{array}$$

is negligible. However, we have to be delicate here. For example, if the adversary wants to tamper with *W*, he has access to *L* and knows that $\mathrm{NMDec}\left(\underline{L},\underline{R}\right)=\mathrm{NMDec}\left(L,R\right)$. This reveals some information about *R* and thus the adversary potentially might get hold of some partial information about the encoded data (and $K_{w},K_{c}$ in particular). This is why it is actually easier to directly argue that

(3) $$\begin{array}{c} \mathrm{Pr}(\mathrm{tampered}\;\mathrm{codeword}\;\mathrm{is}\;\mathrm{valid}\;\wedge \left(\underline{W},\underline{C}\right)\neq \left(W,C\right)\wedge \mathrm{NMDec}\left(\underline{L},\underline{R}\right)=\mathrm{NMDec}\left(L,R\right)) \end{array}$$

is negligible. Notice that the codeword will not be valid in only one of three cases: if $\mathrm{NMDec}(\underline{L},\underline{R})=⊥$ or if one of the MACs on *W* or *C* does not verify correctly. Since $\mathrm{NMDec}\left(\underline{L},\underline{R}\right)=\mathrm{NMDec}\left(L,R\right)$, we know that the only options left are the failures to verify MACs. Moreover, we know that $\left(\underline{K_{w}},\underline{K_{c}}\right)=\left(K_{w},K_{c}\right)$, thus Inequality (3) can be rewritten:

(4) $$\begin{array}{c} \mathrm{Pr}({\mathrm{Vrfy}}_{K_{w}}\left(W,\sigma_{w}\right)={\mathrm{Vrfy}}_{K_{c}}\left(C,\sigma_{c}\right)=1\wedge \left(\underline{W},\underline{C}\right)\neq \left(W,C\right)\wedge \mathrm{NMDec}\left(\underline{L},\underline{R}\right)=\mathrm{NMDec}\left(L,R\right)) \end{array}$$

is negligible. Now, we can upper-bound the term in the Inequality (4) by the following

$$\begin{array}{c} \mathrm{Pr}({\mathrm{Vrfy}}_{K_{w}}\left(W,\sigma_{w}\right)={\mathrm{Vrfy}}_{K_{c}}\left(C,\sigma_{c}\right)=1\wedge \left(\underline{W},\underline{C}\right)\neq \left(W,C\right)), \end{array}$$

which by the union bound can be upper-bounded with

$$\begin{array}{c} \mathrm{Pr}\left({\mathrm{Vrfy}}_{K_{w}}\left(W,\sigma_{w}\right)=1\wedge \underline{W}\neq W\right)+\mathrm{Pr}\left({\mathrm{Vrfy}}_{K_{c}}\left(C,\sigma_{c}\right)=1\wedge \underline{C}\neq C\right). \end{array}$$

Finally, we can argue that each of the elements of the sum is negligible. Notice that when tampering with a *W* adversary has access to *L* but that can not reveal any information about $K_{w}$ since every non-malleable code is a secret sharing scheme. The rest follows from the security of MACs.

#### 6.2.2. Codeword Is in $P_{3}$ i.e., $\mathrm{NMDec}\left(\underline{L},\underline{R}\right)\neq \mathrm{NMDec}\left(L,R\right)$

In this case, we will follow the adventures of the seed *S*; the MACs and keys do not play any role here. In fact, for the purposes of this proof sketch, we will ignore the MAC keys and tags. We will also assume that this case (i.e., codeword $\in P_{3}$) occurs with substantial probability (else we do not have to worry about it). In such a case, we will argue that the final message is independent of the original message.

We start with replacing *C* (see Figure 2) with $\tilde{C}$ where $\tilde{C}$ is completely uniform and independent of the message (eventually, we would need to replace $\tilde{C}$ back with C=Ext(W,S)⊕m).

After technical transformations, the authors obtain that:

(5) $$\mathrm{Ext}\left(W;S\right)\approx U|S,L,\tilde{C},\underline{L},\mathrm{Ext}\left(\underline{W};\underline{S}\right),\underline{S}$$

The intuition behind the equation above is very similar to the case of $P_{rest}$ in Section 6.1; the proof is more involved than the one in [33], but we omit the technical details behind Equation (5). In addition, note that, in the equation above, there is no dependence on *m* on either side as $\tilde{C}$ is independent of *m*. Ultimately, we would like to say that the output of the tampering experiment is indistinguishable from the simulated output. The authors accomplish this in three steps:

1. Adding *R*

In Equation (5), the only information correlated to *W* and *R* is $\underline{S}$. Since Ext(W;S)≈U even given $\underline{S}$, we can safely add *R* to Equation (5).

$$\mathrm{Ext}\left(W;S\right)\approx U|S,L,\tilde{C},\underline{L},\mathrm{Ext}\left(\underline{W};\underline{S}\right),\underline{S},R,g_{1}\left(R,\tilde{C},{\tilde{\sigma }}_{c}\right)\;.$$

From here, we would ideally like to drop $\tilde{C}$ and somehow bring back the dependence on *m* via *C*. For now, we drop $\tilde{C}$

(6) $$\mathrm{Ext}\left(W;S\right)\approx U|S,L,\underline{L},\mathrm{Ext}\left(\underline{W};\underline{S}\right),\underline{S},R,\underline{R}\;.$$

The way, we will bring *C* is to condition $\tilde{C}$ on being a “cipher of *m*”. For that, we first need to prove that $\tilde{C}$ is independent of *W* given appropriate auxiliary information.

2. Capturing $\tilde{C}$’s correlation with *W*

In this step, the authors prove that $\tilde{C}$ is independent of *W* given $S,L,\underline{L},\mathrm{Ext}\left(\underline{W};\underline{S}\right),\underline{S},R,\underline{R}$. We first observe that $\tilde{C}$ is independent of *W* given (L,R,S). Now, we would like to add the other random variables in the auxiliary information. The authors use a Lemma due to Dziembowski and Pietrzak which states that independence in the presence of additional auxiliary information is indeed possible, provided it satisfies a few properties:

- The auxiliary information may be computed in multiple steps;
- Computation in all of the steps can use (L,R,S) and the part of the auxiliary information generated in previous steps;
- Computation in a given step can either depend on $\tilde{C}$ or *W* but not both.

By computing auxiliary information in the order $\underline{L}$ followed by $\underline{R}$ followed by $\mathrm{Ext}(\underline{W};\underline{S})$, one can easily prove that $\tilde{C}$ is independent of *W* given $S,L,\underline{L},\mathrm{Ext}\left(\underline{W};\underline{S}\right),\underline{S},R,\underline{R}$.

3. Conditioning $\tilde{C}$ appropriately

Since *W* is independent of $\tilde{C}$ given appropriate auxilliary information, in Equation (6), we can condition $\tilde{C}$ to either be m⊕Ext(W,S) or m⊕U. (Note that the former is identical to *C*.) By doing so, Equation (6) will lead to the following $C,S,L,\underline{L},\mathrm{Ext}\left(W;S\right),\underline{S},R,\underline{R}\approx U,S,L,\underline{L},\mathrm{Ext}\left(W;S\right),\underline{S},R,\underline{R}$, where $\underline{R},\underline{S}$ are appropriately computed.

The desired result follows by observing that the tampered codeword is a function of

$$\underline{L},\underline{R},\mathrm{Ext}\left(\underline{W};\underline{S}\right),C,R\;.$$

Putting It Together

Thus far, we have described the simulator and sketched the proof for showing that the simulated output is indistinguishable from the tampered output in each of the cases. To complete the proof, we need to combine all three cases and, in particular, the probability that the codewords (tampered vs. simulated) lie in each of the partitions needs to be analysed.

To do this, the authors follow a standard argument: they consider a “skewed” codeword, which, like the tampered codeword, encodes the real message. However, the probability with which the skewed codeword lies in various partitions are the same as for the simulated codewords (in other words, “skewed” codeword behaves like original codeword on each partition, but partitions are “assembled” with slightly modified probabilities). The authors complete the proof by showing that the probability that the tampered codeword lies in a partition is independent of the message and then combines all three cases using the skewed codeword as an intermediate hybrid.

This allows the authors to finish the argument about tampered message not revealing the original message.

Candidate Instantiation

**Figure 3:**
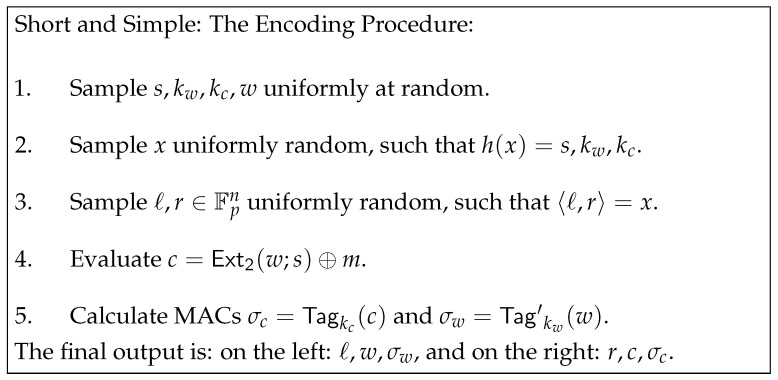
Simple non-malleable code with a great rate. Here, *h* is an affine evasive function. The decoding procedure is analogous: the decoder inverts Steps 3 and 2, obtains keys $k_{w},k_{c}$, verifies MACs from the Step 5 and proceeds to obtain the message via Step 4. If in Step 2 the function *h* outputs ⊥, then the decoder aborts and outputs ⊥.

While one can turn any augmented non-malleable code (or randomness encoder) into a good rate non-malleable code, a very simple result can be obtained using [9]. To encode a message *m*, all we will need is *an affine evasive functionh*. It is a function $h:F_{p}\to M\cup ⊥$ such that Pr(h(aU+b)≠⊥|h(U)=m) is negligible for all a,b,m, and U|h(U)=m should be efficiently sampleable, the construction of the said function can be found in [9,49]. The encoding procedure is described in Figure 3.

## 7. Application: Non-Malleable Codes for Computable Tampering

Split-state tampering functions, even when allowed leakage between the states, are subject to strong independence constraints. In this section, we will look at tampering families without any such constraints but instead having limited *computational complexity*. In fact, we will show, in some sense, how to reduce computational constraints to independence by showing how to construct non-malleable codes for a variety natural computational tampering classes from split state non-malleable codes. We will consider the following tampering classes:

- Decision tree tampering (Section 7.1 [46]): each tampered output symbol is a function of a small polynomial number of (adaptively chosen) queries to codeword symbols.
- Small-depth circuit tampering (Section 7.2 [22,46,47]): the tampered codeword is produced by a boolean circuit of polynomial size and nearly logarithmic depth.
- (Bounded) Polynomial-size circuit tampering (Section 7.3 [48]): the tampered codeword is produced by circuit of bounded polynomial size, $n^{d}$ for some constant *d* where *n* is the codeword length.

On computational complexity and non-malleable codes

We begin with some remarks connecting non-malleable codes with more conventional computational complexity. First, we note that non-malleable codes for circuit classes require circuit lower bounds.

**Proposition** **1** (Informal). *For most natural tampering classes, C, an explicit non-malleable code resilient to tampering by class C implies a circuit lower bound for that class: an explicit function that is hard for C to compute.*

In particular, if (Enc,Dec) is a non-malleable code resilient to C tampering, then Dec cannot be computed by C. Suppose not; then, consider the tampering function that computes Dec and outputs a fixed encoding 0 if the first bit of the message is 1 and outputs a fixed encoding of 1, otherwise. Moreover, it is not difficult to observe that Enc gives rise to (efficiently sampleable) input distributions against which Dec is *hard-on-average* for C to compute.

Given our difficulties in proving circuit lower bounds, one interpretation of this observation is that we can only expect to construct *unconditionally secure* non-malleable codes against very limited circuit classes—or in other words, non-malleable codes for expressive circuit classes, such as polynomial size circuits, require computational assumptions.

Given that (strong) circuit lower bounds are necessary for non-malleable codes, one might wonder if they are sufficient. In general, this is not true.

**Theorem** **15** (Informal [60]). *Explicit hard functions for a class C do not imply non-malleable codes for C.*

Consider the class of tampering functions, ${\mathrm{Local}}^{n-1}=\left\{f\right\}$, such that each output bit is an arbitrary function of all but 1 of the input bits, i.e., for each j∈[n] the function computing the *j*th tampered bit, $f_{j}$ can be written as $f_{j}\left(c_{1},c_{2},\ldots ,c_{i_{j}-1},c_{i_{j}+1},\ldots ,c_{n}\right)$ for some $i_{j}\in \left[n\right]$. It is easy to observe that such functions cannot compute Parity, i.e ${⊕}_{i}c_{i}$. (There is a syntactic problem here in that the output length of Parity does not match that of the tampering class, but consider instead a function whose first bit of output is the Parity of its inputs.) In fact, functions in ${\mathrm{Local}}^{n-1}$ have *no* advantage over random guessing computing Parity of uniformly random inputs.

One might hope to use the fact that Parity is hard for this class, ${\mathrm{Local}}^{n-1}$, directly by encoding a single bit *b* as uniformly bits $c_{1},\ldots ,c_{n}$ such that ${⊕}_{i}c_{i}=b$. However, note that this code, while providing some form of leakage-resilience, is trivially malleable by the class: consider the function that flips the first bit.

This straw man argument intuitively leads us to believe that non-malleability requires much more than (average-case) circuit lower bounds. Ref. [60] justified this intuition, proving that non-malleable codes for ${\mathrm{Local}}^{n-1}$ tampering *do not exist*.

Key Idea: Communication Bottlenecks

We saw that the straw man approach of encoding directly using a hard function for a computational tampering class will not succeed; instead, we show how to leverage split-state non-malleable codes to construct non-malleable codes against computational tampering classes. The high level intuition for all of these constructions is to induce and exploit *communication bottlenecks* in the tampering computation.

What do we mean by communication bottlenecks? Imagine that the (random) inputs to a computation can be partitioned into two subsets *X* and *Y* such that two parties, Alice (holding *X*) and Bob (holding *Y*), can simulate the computation by communicating at most *t* bits. Why is this helpful? This class of computation (independent tampering on *X* and *Y* conditioned on small communication between *X* and *Y*) precisely corresponds to the tampering class handled by (adaptive) leakage-leakage resilient split-state non-malleable codes (See extensions to Definition 7 and Remark 1). For clarity, we define this tampering class as two-party *t*-communication tampering (This class is also referred to as “leaky split state tampering” in the literature. ).

**Definition** **14.** *Let $f:{\left\{0,1\right\}}^{n}\times {\left\{0,1\right\}}^{n}\to {\left\{0,1\right\}}^{n}\times {\left\{0,1\right\}}^{n}$ be a function and $f_{A}:{\left\{0,1\right\}}^{n}\to {\left\{0,1\right\}}^{n}$,$f_{B}:{\left\{0,1\right\}}^{n}\to {\left\{0,1\right\}}^{n}$ such that $f\left(x,y\right)=f_{A}\left(x,y\right),f_{B}\left(x,y\right)$.*

*We say that f is a * two-party t(n)-communication tampering function*if there is a two-party protocol $\Pi_{f}$ where two parties Alice and Bob communicate at most t(n) bits such that, for any $x,y\in {\left\{0,1\right\}}^{n}$, if Alice is given x and Bob is given y, Alice outputs $f_{A}\left(x,y\right)$ and Bob outputs $f_{B}\left(x,y\right)$.*

*We denote the class of two-party t(n)-communication tampering functions as t(n)−SS. Moreover, we say a non-malleable code for this tampering class is *augmented* if the left half of the codeword, communication transcript, and outcome of the tampering experiment can be jointly simulated.*

Our goal, in this section, is to construct coding schemes, Enc,Dec that induce communication bottlenecks when composed with any tampering function in the target class, i.e., for any tampering *f*, the function Dec(f(Enc(X,Y)) can be simulated by a two party protocol with at most *t* bits of communication (existing leakage-resilient split-state codes can handle *t* that is a constant fraction of |X| and |Y|). More precisely, we want to construct a non-malleable reduction (Definition 4), Enc,Dec, from the computational tampering class, C, to two-party *t*-communication tampering, i.e., for every tampering function f∈C there exists some distribution $D_{f}$ over two-party *t*-communication tampering protocols such that

$$\mathrm{Dec}(f\left(\mathrm{Enc}\left(X,Y\right)\right)\approx_{\varepsilon }D_{f}\left(X,Y\right).$$

In Section 7.1, we will see how [46] constructs such a non-malleable reduction for the class of decision tree tampering functions: where each tampered output bit is produced by a bounded number of queries to the input bits. Ref. ([46]’s construction extends an earlier of [61] for local tampering functions, which corresponds to the case where the queries are static: chosen independently of the input.)

Section 7.2 will not construct a communication bottlenecking non-malleable reduction directly, but implicitly. In particular, this section will present [47]’s non-malleable reduction from small-depth circuit tampering to decision tree tampering. This reduction, as well as an earlier (but inefficient) construction of [22], critically uses a technique from the circuit lower bound literature: random restrictions.

Finally, in Section 7.3, we will see how assumptions in the derandomization literature can be used to induce communication bottlenecks in polynomial size circuit tampering. In particular, how Ref. [48] uses hardness against *nondeterministic* circuits to construct non-malleable codes for polynomial size circuit tampering from augmented leakage-resilient split-state non-malleable codes. The code presented here has only inverse polynomial security. Other constructions for this class are known that do not rely on split-state non-malleable codes. Unfortunately, while these constructions are beautiful and achieve negligible security error, they are not fully explicit: relying either on an untamperable common random strings (CRS model) [10,62], or poorly understood heuristic cryptographic assumptions [63,64]. (The latter constructions from cryptographic assumptions only achieve computational security: no *efficient* distinguisher (polynomial size circuit) can distinguish the real and simulated experiments).

Challenges (Comparison with Pseudorandomness)

The idea of communication bottlenecks has a fruitful history in pseudorandomness [65,66,67,68], but our setting presents unique challenges that make it difficult to extend results directly.

Firstly, non-malleable *codes* are required to meaningfully encode (and decode) information. (In contrast, pseudorandomness is only required to “fool” the computation.) While it is often intuitive how to tweak a pseudorandom generator to encode information, we must also simulate decoding of whatever the computation outputs with low communication, which can be delicate as the adversarial tampering could try to force decoding to behave badly.

Secondly, and perhaps more importantly, non-malleable codes must handle adversarial computations that take *n* bits of input and output *n* bits. (Compare with pseudorandomness, where it only necessary (and possible) to consider adversarial computations with short output.) For example, while it is straightforward to fool a single decision-tree (using bounded-inependence), *n* decision trees can copy *X* to the *Y* portion which cannot be simulated with low communication.

On the upside, here the adversarial computation does not have the last word: the (standard) non-malleability experiment only outputs after decoding. Additionally, non-malleable codes are not concerned with pseudorandomness, so there is no need to stringently account for the randomness consumed by the encoding.

Despite these differences, some of the constructions here will draw on techniques from the pseudorandomness literature, particularly those of Section 7.2 and Section 7.3.

### 7.1. Decision Tree Tampering

Now we will give an overview of [46]. As mentioned above, decision trees of depth *d* capture tampering where each output bit is set arbitrarily after adaptively reading *d* locations of the input, where the choice of which input location to read next at any point in time can depend on the values of all the previous locations read.

**Definition** **15** (Decision Trees)**.** *A decision tree with n input bits is a binary tree whose internal nodes have labels from $x_{1},\cdots ,x_{n}$ and whose leaves have labels from {0,1}. If a node has label $x_{i}$, then the test performed at that node is to examine the i-th bit of the input. If the result is 0, one descends into the left subtree, whereas, if the result is 1, one descends into the right subtree. The label of the leaf so reached is the output value on that particular input. The *depth* of a decision tree is the number of edges in a longest path from the root to a leaf. Let DT(t) denote decision trees with depth at most t.*

Ref. [46] constructs non-malleable codes resilient to tampering by decision-trees of depth $n^{1/4-o(1)}$.

**Theorem** **16** ([46]). *For any $t=O(n^{1/4}/\log^{3/2}n)$, there is an explicit and efficient non-malleable code that is unconditionally secure against depth-t decision trees with codeword length $n=O(kt^{2}\log^{4}n/\log\log n)$ and error $\exp(-\Omega \left(n/t^{4}\log^{5}n\right))$ for a k-bit message.*

Technical Overview

This theorem follows by constructing a non-malleable reduction (Definition 4) from decision-tree tampering to two-party bounded communication tampering. Theorem 16 follows from composing this reduction with a leakage-resilient split-state non-malleable code (i.e., a non-malleable code for two-party bounded communication tampering).

**Lemma** **13** (NMR from [46]). *For any constant α∈(0,1) and $t=O(n^{1/4}/\log^{3/2}n)$, there is a (DT(t)⇒t(n)−SS,ε)-non-malleable reduction with rate $\Omega (1/t^{2}\log^{3}n)$ where ε≤$\exp(-\Omega \left(n/t^{4}\log^{5}n\right))$.*

We will outline [46]’s reduction for decision tree tampering. Their reduction builds on a reduction of [61] for local tampering (where the bounded number queries to codeword are made non-adaptively). In fact, the two reductions are quite similar (though not identical); however, the analysis differs substantially.

The key idea of this construction is to exploit size differences. The encoder and decoder will work independently on the left and right pieces of the message, so we will in turn think of having left and right encoders, decoders, codewords, and tampering functions (corresponding to the respective outputs).

First, suppose that the right piece of the message (corresponding to the right split-state codeword) is much longer than that of the left. Then, suppose both the right and left encoders and decoders are simply the identity function. Then, all the left tampering functions together will make a number of queries to the right codeword that is below the leakage threshold.

However, because the right is much longer than the left, the above analysis will not help in simulating tampering on the right with low leakage from the left. Instead, Ref. [46] modifies the left encoder/decoder to make it much longer than the right, but while retaining the property that the left can be decoded from just a few decision trees. To do so, sample a random small set, whose size is that of the message, in a much larger array. Then, plant the message in these locations and zero everything else out. Then, the bit-wise secret shares a description of the small set (i.e., its seed) such that the secrecy threshold is relatively large. To decode, simply extract the seed and output what is in the corresponding locations of the array.

Now, note that decoding the left still only requires at most relatively few queries to the right: decision tree depth times both encoded seed length plus message length. However, we can not make the encoded seed too long or we will be dead again. Instead, Ref. [46] critically uses the fact that tampering is by a *forest* of decision trees. In particular, for any small set of tampering functions on the right, the seed remains uniformly chosen regardless of what queries the set makes, so we expect only a small fraction of any queries made to the array to actually hit the message locations. Strong concentrations bounds guarantee that this is more or less what actually happens. Then, simply union bound over all such subsets to guarantee that collectively the right tampering function makes few queries to the left with overwhelming probability.

Finally, apply the same style of encoding used on the left to the right side to fix the syntactic mismatch and reduce to the case where the right and left messages are the same size.

### 7.2. Small-Depth Circuit Tampering

We will give an overview of [47]. Small-depth circuit tampering captures the case where each tampered output bit is produced by a size $S\left(n\right)=n^{\omega (1)}$ circuit of depth d(n)=o(logn/loglogn) over the standard basis with arbitrary fan-in, we denote this class ${\mathrm{AC}}_{d}\left(S\left(n\right)\right)$. (This includes the case where each output is produced by a constant depth polynomial size circuit, ${\mathrm{AC}}^{0}$.)

**Theorem** **17** ([46,47]). *For $d\leq c_{1}\log n/\log\log n$, there exists an explicit, efficient, information theoretic non-malleable code for d-depth circuits (of unbounded fan-in) of size $\exp(n^{c_{2}/d})$ with error $\exp(-n^{\Omega (1/d)})$ and encoding length $n=k^{1+c}$, where $c,c_{1},c_{2}\in \left(0,1\right)$ are constants.*

*For the special case of ${\mathrm{AC}}^{0}$-tampering, there exist efficient non-malleable codes for O(1)-depth polynomial size circuits circuits with negligible error and encoding length $n=k^{1+o(1)}$.*

Ref. [47] showed how to use a tool from circuit lower bounds and derandomization, pseudorandom switching lemmas, to construct a non-malleable reduction from small-depth circuit tampering to decision tree tampering (which, as we have seen, can be reduced to split-state tampering). Prior to this construction, Chattopadhyay and Li had constructed an (invertible) *seedless non-malleable extractor* for small-depth circuit tampering [22]. However, unlike the construction here, the error of their extractor yields inefficient non-malleable codes (*k* length messages encode into codewords of length $n=2^{\Omega (\sqrt{k})}$). We state [47]’s main technical lemma, a non-malleable reduction from small-depth circuit tampering to small-depth decision tree tampering, before sketching their non-malleable reduction and its analysis here.

**Lemma** **14** ([47]). *For S,d,n,t∈N,p,δ∈(0,1), there exists $\sigma =\mathrm{poly}(t,\log\left(2^{t}S\right),\log\left(1/\delta \right),$log(1/p)) and m=O(σlogn) such that, for any $2m\leq k\leq n{\left(p/4\right)}^{d}$,*

$$\left({\mathrm{AC}}_{d}\left(S\right)\Rightarrow \mathrm{DT}\left(dmt\right),\varepsilon \right)$$

*where*

$$\varepsilon =nS\left(2^{2t+1}{\left(5pt\right)}^{t}+\delta \right)+\exp\left(-\frac{\sigma }{2\log(1/p)}\right).$$

Let us start by considering the simpler case of reducing *w*-DNFs (each clause contains at most *w* literals) to low-depth decision tree tampering. The reduction for general small-depth circuits will follow from a recursive composition of this reduction.

A non-malleable reduction (E,D) reducing DNF-tampering to small-depth decision tree tampering needs to satisfy two conditions (i) Pr[D(E(x))=x]=1 for any *x* and, (ii) D∘f∘E is a distribution over small-depth decision trees for any width-*w* DNF *f*. A classic result from circuit complexity, the switching lemma [69,70,71,72] states that DNFs become small-depth decision trees under random restrictions (“killing” input variables by independently fixing them to a random value with some probability). Thus, a natural choice of E for satisfying (ii) is to simply sample from the generating distribution of restrictions and embed the message in the surviving variable locations (fixing the rest according to restriction). However, although f∘E becomes a decision tree, it is not at all clear how to decode and fails even (i). To satisfy (i), a naive idea is to simply append the “survivor” location information to the encoding. However, this is now far from a random restriction (which requires among other things that the surviving variables are chosen independently of the random values used to fix the killed variables) is no longer guaranteed to “switch” the DNFs to decision trees with overwhelming probability.

To circumvent those limitations, we consider *pseudorandom switching lemmas*, usually arising in the context of derandomization [73,74,75,76,77,78], to relax the stringent properties of the distribution of random restrictions needed for classical switching lemmas. In particular, we invoke a pseudorandom switching lemma from Trevisan and Xue [77], which reduces DNFs to decision trees while only requiring that randomness specifying survivors and fixed values be σ-wise independent. This allows us to avoid problems with independence arising in the naive solution above. Now, we can append a σ-wise independent encoding of the (short) random seed that specifies the surviving variables. This gives us a generating distribution of random restrictions such that (a) DNFs are switched to decision trees, and (b) the seed can be decoded and used to extract the input locations.

At this point, we can satisfy (i) easily: D decodes the seed (whose encoding is always in, say, the first *m* coordinates), then uses the seed to specify the surviving variable locations and extract the original message. In addition to correctness, f∘E becomes a distribution over local functions where the distribution only depends on *f* (not the message). However, composing D with f∘E induces dependence on underlying message: tampered encoding of the seed may depend on the message in the survivor locations. The encoded seed is comparatively small and thus (assuming the restricted DNF collapses to a low-depth decision tree) requires a comparatively small number of bits to be leaked from the message in order to simulate the tampering of the encoded seed; given a well simulated seed, we can accurately specify the decision trees that will tamper the input (the restricted DNFs whose output locations coincide with the survivors specified by the tampered seed). This intermediate leaky decision tampering class, which can be described via the following adversarial game: (1) the adversary commits to *N* decision trees, (2) the adversary can select *m* of the decision trees to get leakage from, and (3) the adversary then selects the actual tampering function to apply from the remaining local functions. However, provided the seed length, *m*, is short enough, this just amounts to querying a slightly higher depth decision tree.

To deal with depth *d* circuits, we can recursively apply this restriction-embedding scheme *d* times. Each recursive application allows us to trade a layer of gates for another (adaptive) round of *m* bits of leakage in the leaky decision tree game. One can think of the recursively composed simulator as applying the composed random restrictions to collapse the circuit to decision trees and then, working inwardly, sampling all the seeds and the corresponding survivor locations until the final survivor locations can be used to specify ultimate decision tree tampering.

### 7.3. (Bounded) Polynomial Size Circuit Tampering

In this subsection we follow [48], we show how to construct non-malleable codes for tampering by $n^{c}$-size circuits, where *c* is some constant. As mentioned at the outset, non-malleable codes for circuit tampering imply circuit lower bounds. Given that explicit lower bounds against superlinear size circuits are well-beyond our current techniques in complexity, assumptions are needed for such non-malleable codes. Ref. [48] showed how to use hardness assumptions against *nondeterministic* circuits to construct such codes from split-state non-malleable codes. We begin by presenting the hardness assumption before giving a brief overview of [48]’s construction.

**Definition** **16** (Nondeterministic circuit). *A* nondeterministic circuit* C is a circuit with “non-deterministic” inputs, in addition to the usual inputs. We say C evaluates to 1 on x if and only if there exists an assignment, w, to the non-deterministic input wires such that the circuit, evaluated deterministically on input (x,w) outputs 1.*

**Assumption** **1** (E requires exponential size nondeterministic circuits). *There is a language $L\in E=\mathrm{DTIME}(2^{O(n)})$ and a constant γ such that, for sufficiently large n, non-deterministic circuits of size $2^{\gamma n}$ fail to decide L on inputs of length n.*

Informally, the above assumption says that non-uniformity and non-determinism do not always imply significant speed-ups of uniform deterministic computations.

**Theorem** **18** ([48]). *If E requires exponential size non-deterministic circuits, then, for every constant c, and for sufficiently large k, there is an explicit, efficient, $n^{-c}$-secure non-malleable code for k-bit messages, with codeword length n=poly(k), resilient to tampering by $n^{c}$-size circuits.*

Ref. [48] constructs their codes by “fooling” non-malleable codes for *split-state tampering* with special properties: augmented, leakage-resilient, and admitting a special form of encoding (given half a codeword can efficiently sample the other half to encode any message).

Split-state tampering functions may manipulate the left and right halves of a codeword arbitrarily, but independently (i.e., functions such that $\left(c_{L},c_{R}\right)\mapsto \left(f_{L}\left(c_{L}\right),f_{R}\left(c_{R}\right)\right)$ for some $f_{L},f_{R}$). Leakage-resilient split-state tampering allows each tampered codeword half to depend on bounded leakage from the opposite codeword half. In addition to split-state NMC, Ref. [48] also uses a pseudorandom generator (PRG) for nondeterministic circuits, where $c^{\prime }>c$ is a constant. In particular, they require that the PRG, *G*, is secure even when given the seed (seed extending), i.e., no nondeterministic circuit of bounded polynomial size can distinguish G(s) from a uniformly random string *and* *s* is a prefix of G(s). The existence of such PRGs follows from Assumption A1 [79,80,81,82,83,84].

Given a (leakage-resilient) split-state non-malleable code, with necessary properties and a seed-extending pseudorandom PRG for nondeterministic circuits, *G*, we encode a message *x* by sampling the following:

$$\left(s,c_{R}\right)\mathrm{such}\mathrm{that}\left(G\left(s\right),c_{R}\right)\mathrm{is}\;a\;\mathrm{split}-\mathrm{state}\;\mathrm{encoding}\;\mathrm{of}\;x.$$

The proof proceeds by contradiction starting with the assumption that the construction is not non-malleable. The analysis follows by giving a nondeterministic reduction that uses the (assumed) malleablity of the construction to violate the PRG security.

1. Assume towards contradiction that $\left(s,c_{R}\right)$ is *malleable* and fix the corresponding poly-size tampering function *g*, which is *not* split-state and violates non-malleability.
2. Transform *g* into a split-state tampering function $f_{L},f_{R}$ on $\left(c_{L},c_{R}\right)$, where (1) $f_{L}$ is *unbounded*, relies on |s| bits of leakage from $c_{R}$ and returns some $c_{L}^{\prime }$, (2) $f_{R}$ is efficient, relies on |s| bits of leakage from $c_{L}$ and returns $c_{R}^{\prime }$. Crucially, a split-state tampering function $\left(f_{L},f_{R}\right)$ is guaranteed to break non-malleability when $c_{L}=\left(s||y\right)=G\left(s\right)$.
3. Since $\left(c_{L},c_{R}\right)$ is a leakage-resilient split-state non-malleable code where $c_{L}$ is uniform random, then when $c_{L}$ is random (as opposed to in the construction where codewords are sampled as $\left(G\left(s\right),c_{R}\right)$), every tampering functon $\left(f_{L}^{\prime },f_{R}\right)$ *fails* to break non-malleability, even when $f_{L}^{\prime }$ is unbounded and chooses its output $c_{L}^{\prime }$ in the “optimal” way.
4. Construct an Arthur–Merlin protocol (with bounded poly-size Arthur), that distinguishes between input $c_{L}$ being random or pseudorandom. Such a protocol can then be transformed into a non-deterministic polynomial bounded circuit (this follows from classical results: IP[O(1)]⊆AM⊆NP/poly [84,85,86,87]).
5. Intuitively, Arthur can efficiently compute all the values needed to simulate the tampering experiment except for $c_{L}^{\prime }$, which is obtained from Merlin. Specifically, on input $c_{L}$, Arthur samples $c_{R}$, and computes $c_{R}^{\prime }=f_{R}\left(c_{R}\right)$, as well as the leakage on $c_{R}$. Arthur sends $c_{L}$ and the leakage on $c_{R}$ to Merlin who responds with $c_{L}^{\prime }$. If $c_{L}$ is pseudorandom, then an honest Merlin will return $c_{L}^{\prime }=f_{L}\left(c_{L}\right)$, and, with Merlin’s help, Arthur can check that non-malleability is violated with this $c_{L}^{\prime }$. If $c_{L}$ is random, then, despite any response $c_{L}^{\prime }=f_{L}^{\prime }\left(c_{L}\right)$ from Merlin, non-malleability will *not* be violated, and a dishonest Merlin cannot convince Arthur otherwise.

## 8. Application to Non-Malleable Commitments

In this section, we discuss one of the most important applications of non-malleable codes in the split-state model. In [35], the authors construct a 1/3-rate NMC (which we described in Section 6.2), and then use the textbook non-malleable commitment scheme with computational binding and statistical hiding from [39]. This construction achieves a communication cost of approximately 41 times the length of the message being committed; we begin by defining non-malleable commitments, introduced by Dolev, Dwork, and Naor [88] that give computational binding and statistical hiding property.

**Definition** **17.** *Ref. [39] A non-malleable commitment scheme, 〈C,R〉 is a two-phase, two-party protocol between a committer C and a receiver R. In the commit phase, C uses secret m and interacts with R who uses no input. Let z=Com(m;r) denote R’s view after the commit phase. Let (w,m)=Decom(z,m,r) denote R’s view after the decommit phase, which R either accepts or rejects. We say that 〈C,R〉 is a computationally binding and ε-statistically hiding non-malleable commitment scheme if the following properties hold:*

1. ***Correctness:*** *If the parties follow the protocol, then R(z,w,m)=1, i.e., the receiver accepts.*
2. ***Binding:*** For any PPT adversarial receiver $R^{*}$ that outputs $\left(w^{\prime },m^{\prime }\right),\left(w,m\right),z$, with $m^{\prime }\neq m$, the probability that $R\left(z,w,m\right)=1=R\left(z,w^{\prime },m^{\prime }\right)$ is negligible.
3. ***Hiding:*** *For all distinct message pairs $m,m^{\prime }$, ${\left\{\mathrm{Com}\left(m;r\right)\right\}}_{r}\approx_{\varepsilon }{\left\{\mathrm{Com}\left(m^{\prime };r^{\prime }\right)\right\}}_{r^{\prime }}$.*
4. ***Non-malleability:*** *For avoiding trivial man-in-the-middle attack of copying the identity of the committer, we consider the committer and receiver to additionally have an identity $\mathrm{Id}\in {\left\{0,1\right\}}^{\lambda }$ as common input (λ is the computational security parameter). To define non-malleability, we consider the real/ideal paradigm. In the real interaction, there is a man-in-the-middle adversary M interacting with a committer, C, in the left session and a receiver R in the right. All the quantities associated with the right interaction are denoted by the “tilde’d” versions of their left counterparts (e.g., C commits to m in the left interaction while M commits to $\tilde{m}$ in the right). Let ${\mathrm{MIM}}_{m}$ denote the random variable describing $\left(\mathrm{VIEW},\tilde{m}\right)$, consisting of M’s view in the experiment and the value M commits to in the right interaction, given that C committed to m on the left. The ideal interaction is the same, except that C commits to an arbitrary message, say 0, on the left. Let ${\mathrm{MIM}}_{0}$ denote the corresponding random variable for 0. M is forced to use an identity $\tilde{\mathrm{Id}}$ on the right, which is distinct from Id used on the left. ${\mathrm{MIM}}_{m}$ and ${\mathrm{MIM}}_{0}$ output a special symbol ${⊥}_{\mathrm{Id}}$ when M has used the same identity on the right as received on the left.*
Non-malleability guarantees that, for every PPT man-in-the-middle M, and for all messages m, we have ${\left\{{\mathrm{MIM}}_{m}\left(y\right)\right\}}_{y\in {\left\{0,1\right\}}^{*}}\approx_{c}{\left\{{\mathrm{MIM}}_{0}\left(y\right)\right\}}_{y\in {\left\{0,1\right\}}^{*}}$, where y is the auxiliary input received by M.

*The round complexity of a commitment scheme denotes the number of rounds of interaction between the committer and receiver. The communication complexity of a commitment scheme denotes the total size of the transcript of the interaction between the committer and the receiver.*

The non-malleable commitment scheme from [39] uses a 2-split-state augmented non-malleable code tolerating leakage as an underlying building block. By Theorems 3 and 5, the leakage-resilience requirement can be removed; thus, if one instantiates this scheme with the 1/3-rate augmented non-malleable code, one gets a non-malleable commitment scheme with a communication complexity of 41·|messagelength|. We begin by looking at the building blocks used.

### 8.1. Building Blocks

The construction from [39] requires two building blocks, which were instantiated in [35] as follows.

- A non-interactive computationally binding and statistically hiding commitment, (Com,Decom), with message space ${\left\{0,1\right\}}^{2\beta_{1}}$, which is a non-interactive two phase protocol as in Definition 17 satisfying correctness, computational binding and statistical hiding. There is a hashing based statistically hiding commitment of [89], which has commitment size of ≈9·(messagelength).
- A leakage resilient and augmented non-malleable code, (Enc,Dec), with message space ${\left\{0,1\right\}}^{\alpha }$ and codeword space ${\left\{0,1\right\}}^{\beta_{1}}\times {\left\{0,1\right\}}^{\beta_{2}}$.

### 8.2. Constructions

We now describe the construction of non-malleable commitments from [39], using the building blocks from Section 8.1. For multiplication and addition operations in the construction below, we assume a natural correspondence between the binary $\beta_{1}$-bit strings and the field $GF(2^{\beta_{1}})$.

In [39], the additional property needed from the underlying NMC is called conditional augmented property ([39], [Definition 10]), which guarantees that, if the left state *L* is first picked at random from the space of left state of valid codewords (whose decode is ≠⊥) and then the right state is picked, conditioned on the message and the left state, the augmented non-malleability (with right augmentedness) is still guaranteed; one can observe that the proof of ([39], [Claim 2]), showing that a non-malleable code is conditional augmented, only requires leakage resilience from the left state of the NMC. Hence, the main theorem of ([39], [Theorem 1]), with instantiations from Section 8.1, can be stated as follows.

**Figure 4:**
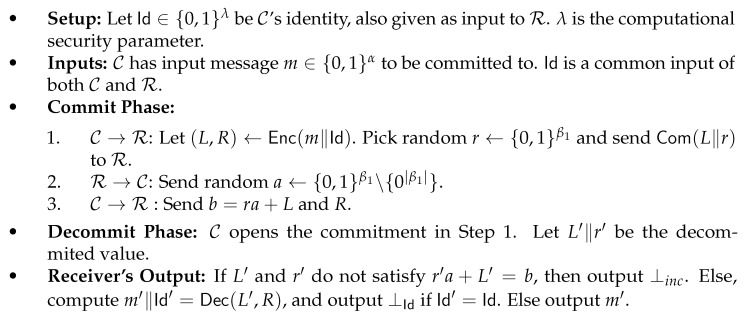
Non-malleable Commitment Scheme 〈C,R〉.

**Theorem** **19.** *Refs. [35,39] If (Com,Decom) is a non-interactive computationally binding and statistically hiding commitment scheme, and (Enc,Dec) is a leakage resilient augmented non-malleable code, then the protocol 〈C,R〉 in Figure 4 is a non-malleable commitment scheme against synchronizing adversary with computational binding and statistical hiding.*

*Furthermore, using the hashing based non-interactive commitment scheme [89] and the non-malleable code from [35], the communication cost of the above scheme is 41 α, where α is the message length.*

## 9. The New Frontier, Open Questions

In this section, we will describe few interesting unresolved questions that are related to this survey:

Exponential error

To date, none of the explicit split-state non-malleable codes achieve genuinely exponential error, $\varepsilon =2^{-\Omega (n)}$. In particular, if one desires error $2^{-k}$, no construction with a codeword of length O(k) is currently known. This is of particular importance in applications where the security parameter may be larger than the message length.

Surpassing Bourgain’s extractor

As we already mentioned in Section 5, further improvements to the constructions of non-malleable extractors would imply an explicit construction of a two-source extractor with a negligible error and low sources entropy requirements. For more reading on the problem, we refer to [90]. Finding such non-malleable extractors is an interesting open question.

Rate above $\frac{1}{3}$

The techniques mentioned in Section 6 do not allow us to build a non-malleable code with a rate better than 1/3. Each of the states has to be at least as long as the message (because of the secret sharing property discussed in Section 3), and the seeded extractor trick requires the length of one of the states to be double the message length. Thus, going below 1/3 requires a new approach, and is left as a (perhaps challenging) open question.

Is the construction from [9] way stronger than we can prove?

We discuss this construction in Section 4. The construction requires a very large size of the vectors ($\Omega (n^{4})$’ coordinates, each of size Ω(n) for an *n*-bit message). We hypothesise, however, that [9] should remain secure even for a constant number of coordinates. Even if the above is not true, finding an explicit attack would greatly expand our understanding of tamper resistance of the inner product. This might even have interesting consequences in additive combinatorics.

Eight is a crowd

As we discuss in Remark 11, the question about the minimum number of states necessary to build a continuous non-malleable code remains unanswered; we know it is at least 3 and at most 8. We hypothesise that extending the techniques from [16] to existential results in non-malleable extractors will yield an existential result in 6-state model. Closing the gap between 3 and 8 would be very interesting, especially if the answer is not 3!

## Figures and Tables

**Table 1:** Prior Work on 2-state NMCs (*n* is codeword length).

Work	Rate
Dziembowski, Pietrzak, Wichs [1]	1/6 (Existential, Random Oracle Model)
Cheraghchi, Guruswami [10]	1/2 (Existential, Lower bound)
Dziembowski, Kazana, Obremski [8]	Ω(1/n) (Only for 1-bit messages)
Aggarwal, Dodis, Lovett, Briët [9,49,50]	$\Omega (1/n^{4/5})$
Chattopadhyay, Goyal, Li [29]	$n^{-\Omega (1)}$
Li [30]	Ω(1/log(n))
Li [31]	Ω(loglog(n)/log(n))
Li [31]	Ω(1) (with constant error)
Aggarwal, Obremski [34]	≈1/1,500,000
Aggarwal, Sekar, Kanukurthi, Obremski, Obbattu [35]	1/3

## Data Availability

Not applicable.
